# Stromal *Pbrm1* mediates chromatin remodeling necessary for embryo implantation in the mouse uterus

**DOI:** 10.1172/JCI174194

**Published:** 2024-03-01

**Authors:** Qiliang Xin, Iris Feng, Guoyun Yu, Jurrien Dean

**Affiliations:** Laboratory of Cellular and Developmental Biology, National Institute of Diabetes and Digestive and Kidney Diseases (NIDDK), NIH, Bethesda, Maryland, USA.

**Keywords:** Endocrinology, Reproductive biology, Epigenetics, Fertility, Sex hormones

## Abstract

Early gestational loss occurs in approximately 20% of all clinically recognized human pregnancies and is an important cause of morbidity. Either embryonic or maternal defects can cause loss, but a functioning and receptive uterine endometrium is crucial for embryo implantation. We report that the switch/sucrose nonfermentable (SWI/SNF) remodeling complex containing polybromo-1 (PBRM1) and Brahma-related gene 1 (BRG1) is essential for implantation of the embryonic blastocyst on the wall of the uterus in mice. Although preimplantation development is unaffected, conditional ablation of *Pbrm1* in uterine stromal cells disrupts progesterone pathways and uterine receptivity. Heart and neural crest derivatives expressed 2 (*Hand2*) encodes a basic helix-loop-helix (bHLH) transcription factor required for embryo implantation. We identify an enhancer of the *Hand2* gene in stromal cells that requires PBRM1 for epigenetic histone modifications/coactivator recruitment and looping with the promoter. In *Pbrm1^cKO^* mice, perturbation of chromatin assembly at the promoter and enhancer sites compromises *Hand2* transcription, adversely affects fibroblast growth factor signaling pathways, prevents normal stromal-epithelial crosstalk, and disrupts embryo implantation. The mutant female mice are infertile and provide insight into potential causes of early pregnancy loss in humans.

## Abstract 2

Early gestational loss is caused by disrupted chromatin remodeling that affects *Hand2* expression and prevents implantation of embryonic blastocysts on the wall of the uterus.

## Graphical abstract

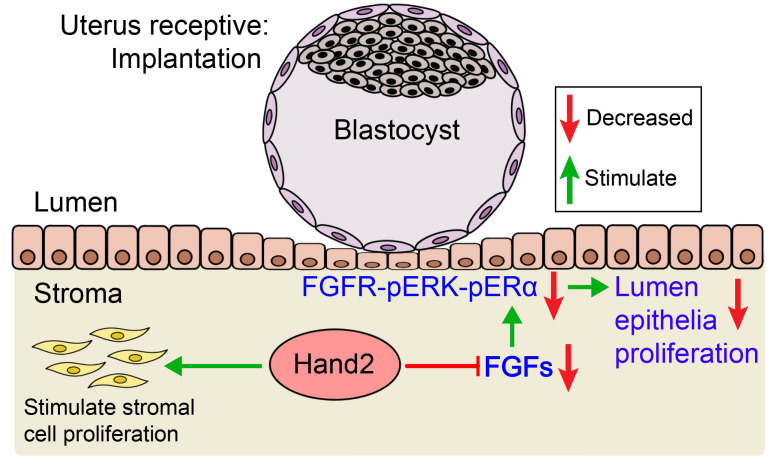

## Introduction

Embryo implantation on the wall of the uterus is a critical step in mammalian reproduction (1). In humans, maximal fecundity for natural births per menstrual cycle is approximately 30%. Over 40% to 50% of conceptions are lost in the first 20 weeks of gestation, and approximately 75% of unsuccessful pregnancies are due to failed implantation (2). Success requires synchronization between a competent embryonic blastocyst and a receptive uterus. There is a temporally restricted “implantation window” regulated by proliferation and differentiation of endometrial epithelium and stroma under the control of progesterone (P_4_) and estrogen (E_2_) (3, 4). Uterine epithelial-stromal crosstalk involves endocrine, paracrine, and juxtacrine interactions that are critical for successful implantation (1, 3). Developmental programs are precisely controlled by chromatin regulators that maintain specific gene expression through epigenetic modification of the genome. However, the details of chromatin remodeling and spatiotemporal expression of genes that guide proper epithelial-stromal interactions to ensure uterine receptivity remain largely unexplored.

As a critical subunit of the switch/sucrose nonfermentable (SWI/SNF) chromatin-remodeling complex PBAF, polybromo-1 -(PBRM1) encodes Brahma-related gene 1–associated (BRG1-associated) factor 180 (BAF180), which (a) targets the complex to specific sites in the genome; (b) recruits additional effector proteins; and (c) alters histone-DNA interactions that control gene expression (5). BRG1 in the PBAF SWI/SNF complex uses energy from ATP hydrolysis to mobilize and reposition nucleosomes at promoters and enhancers. This provides nucleosome-free regions that can accommodate large macromolecular machinery (transcriptional factors, RNA polymerase, etc.) needed to activate target genes (6–8). Using multiple gene-edited mouse lines, molecular biological tools, and bioinformatics, we document that stromal cell expression of *Pbrm1* is required for fertility. By regulating the normal stromal-epithelial dialogue, PBRM1 ensures uterine receptivity and embryo implantation in a SWI/SNF complex–dependent manner. Mechanistically, we determine that PBRM1 is recruited to the *Hand2* uterine enhancer site after P_4_ priming. It promotes *Hand2* transcription by remodeling chromatin accessibility that facilitates transcriptional factor recruitment and enhancer/promoter interactions.

Heart and neural crest derivatives expressed 2 (*Hand2*) encodes a basic helix-loop-helix (bHLH) transcription factor implicated in cardiac morphogenesis and brachial arch development (9–12). More recently, this P_4_-induced gene has been implicated in uterine stromal-epithelial interactions as well as steroid production necessary for embryo implantation (13). *Hand2* expression at the maternal-fetal interface is associated with the evolution of implantation in eutherian mammals as well as gestational regulation that prevents preterm birth (14). However, it remains mostly unknown how initiation of *Hand2* transcription is regulated by epigenetic modifications in uterine stromal cells exposed to P_4_ during embryo implantation.

Enhancers are cis-acting DNA regulatory elements that increase transcriptional output of target genes when brought into proximity of gene promoters through physical interactions by looping of intervening DNA (15, 16). Multiple transcription factors bind enhancers and form integrative hubs to recruit cofactors that bind short, DNA-specific sequences. Enhancers play essential roles in directing cell-, lineage-, and stage-specific gene expression in response to external stimuli (17, 18). Chromatin remodeling for transcription factor binding is a prerequisite for enhancer activity, and chromatin accessibility has been used to identify enhancer sites (19–21). In other tissues, it has been reported that *Hand2* expression is tightly control by upstream enhancers with canonical epigenomic profiles, including H3K4me1 and H3K27ac modifications (10, 11, 22, 23). Thus, we set out to identify enhancers in uterine stromal cells during the “implantation window” that play a molecular role in regulating fertility.

## Results

### Pbrm1 deficiency in the uterus prevents embryo implantation.

**Figure 1:**
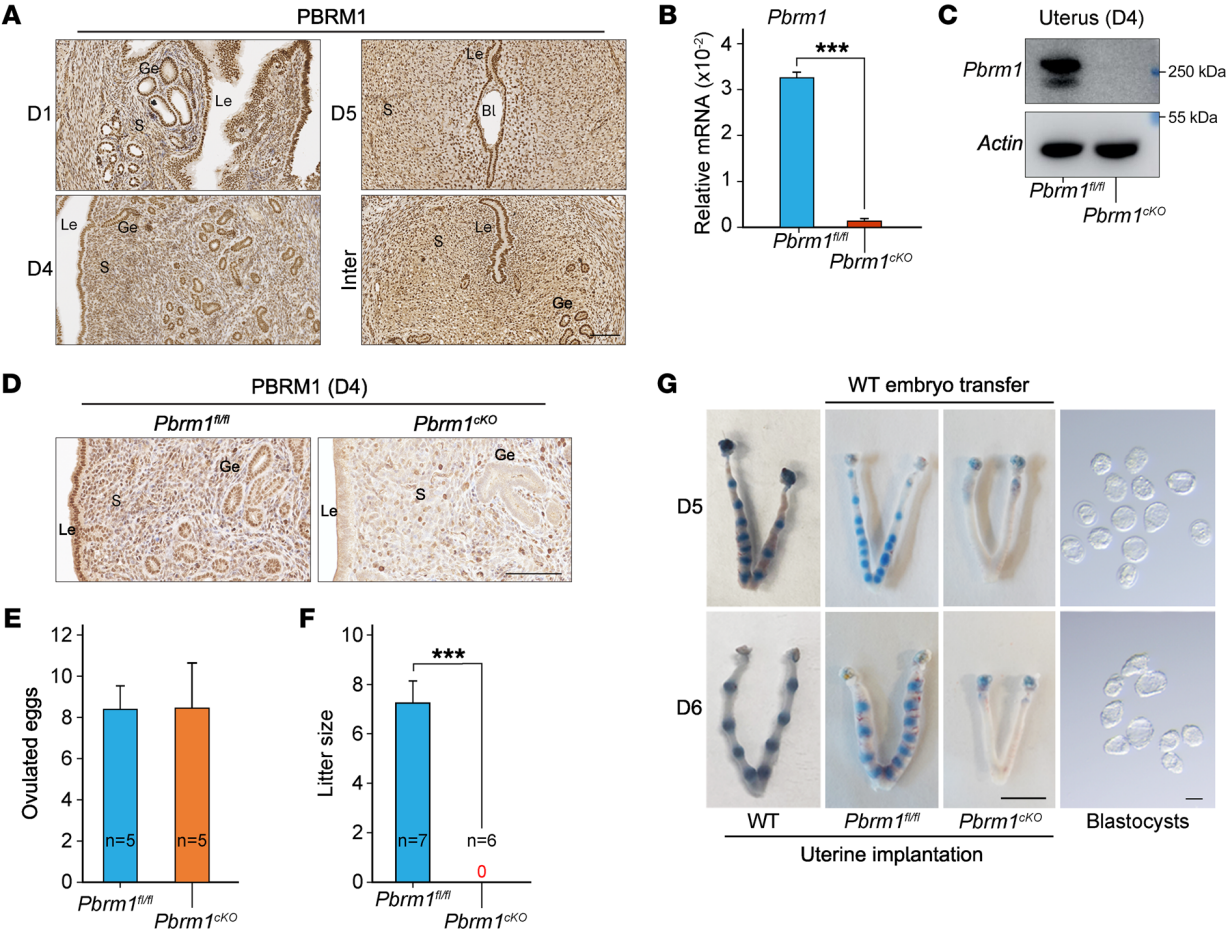
Implantation failure and female infertility after uterine-specific *Pbrm1* deletion. (**A**) Immunohistochemical staining showing the spatiotemporal expression of PBRM1 in WT uteri on D1, D4, and D5 of pregnancy. Bl, blastocyst; S, stromal. Scale bar: 100 mm. (**B**) RT-qPCR analysis of *Pbrm1* mRNA levels in D4 uteri from *Pbrm1^fl/fl^* and *Pbrm1^cKO^* mice. Values are normalized to *Gapdh* expression and represented as mean ± SEM (*n* = 3). ***P < 0.001, independent-sample Student’s *t* test. (**C**) Immunoblotting of PBRM1 protein in D4 *Pbrm1^fl/fl^* and *Pbrm1^cKO^* uteri. β-actin, load control. (**D**) Immunohistochemistry of PBRM1 protein in D4 *Pbrm1^fl/fl^* and *Pbrm1^cKO^* uteri. Scale bar: 100 mm. (**E**) Number of ovulated eggs in *Pbrm1^fl/fl^* and *Pbrm1^cKO^* mice. Numbers within the bars indicate numbers of mice tested. (**F**) Average litter sizes of *Pbrm1^fl/fl^* and *Pbrm1^cKO^* female mice. Numbers within the bars indicate numbers of mice tested. Data are represented as mean ± SEM. ***P < 0.001, independent-sample Student’s *t* test. (**G**) Representative images of normal embryo morphology from D5 to D6 pregnant *Pbrm1^cKO^* mice, exhibiting embryo implantation failure in the uteri beyond D5. Scale bars: 1 cm (uterus); 100 mm (embryos).

To investigate the physiological role of PBRM1 during early pregnancy, we examined its temporal and spatial expression in peri-implantation uteri. Immunohistochemistry documented ubiquitous PBRM1 expression in uterine stroma, glandular epithelium (Ge), and luminal epithelium (Le) during day 1 (D1), D4, and D5 of pregnancy (Figure 1A). The P_4_ receptor (PR) is expressed ubiquitously in the female reproductive tract (24). To investigate the uterine function of PBRM1 during peri-implantation, conditional deletion of *Pbrm1* (*Pbrm1^fl/fl^/PR^IRES-cre/+^*) in the uterus was obtained by crossing *Pbrm1^loxP/loxP^* (*Pbrm1^fl/fl^*) mice with *PR-IRES-cre* (*PR^IRES-cre/+^*) to establish *Pbrm1^cKO^* gene-edited mice. PBRM1 was effectively deleted in *PR*-expressing uterine cells, and the knockout efficacy in the uterus was confirmed by quantitative real-time PCR (RT-qPCR), immunoblot, and immunohistochemistry (Figure 1, B–D).

To determine the importance of PBRM1 in embryo implantation, *Pbrm1^cKO^* mice and their littermate controls (*Pbrm1^fl/fl^*) were mated with fertile WT males. Both *Pbrm1^fl/fl^* and *Pbrm1^cKO^* female mice ovulated a normal complement of eggs (Figure 1E), but the litter size was dramatically reduced to zero in *Pbrm1^cKO^* female mice (Figure 1F). Conditional disruption of *Pbrm1* in the oviduct prevents embryo transport through the female reproductive tract after natural mating (25). Therefore, to determine whether PBRM1 is also required for uterine embryo implantation, WT blastocysts were transferred into WT, *Pbrm1^fl/fl^*, and *Pbrm1^cKO^* pseudopregnant female mice on D4 of pregnancy and analyzed on D5 and D6. Morphologically normal blastocysts were flushed from *Pbrm1^cKO^* uteri at D5 and D6. However, intravenous injection of Chicago Sky Blue dye into *Pbrm1^cKO^* mice did not detect implantation sites marked by the discrete blue dye bands observed in WT and *Pbrm1^fl/fl^* uteri (Figure 1G and Supplemental Table 1; supplemental material available online with this article; https://doi.org/10.1172/JCI174194DS1). Shortly after embryo implantation, uterine stromal cells that surround the blastocyst undergo decidualization that is essential for normal pregnancy (3). Employing an oil-induced decidualization assay (26), the *Pbrm1* mutant uteri showed a remarkably reduced decidual response (Supplemental Figure 1, A and B). Therefore, we conclude that *Pbrm1* is required for normal embryo implantation and uterine decidualization.

### Deletion of Pbrm1 derails normal uterine receptivity.

**Figure 2:**
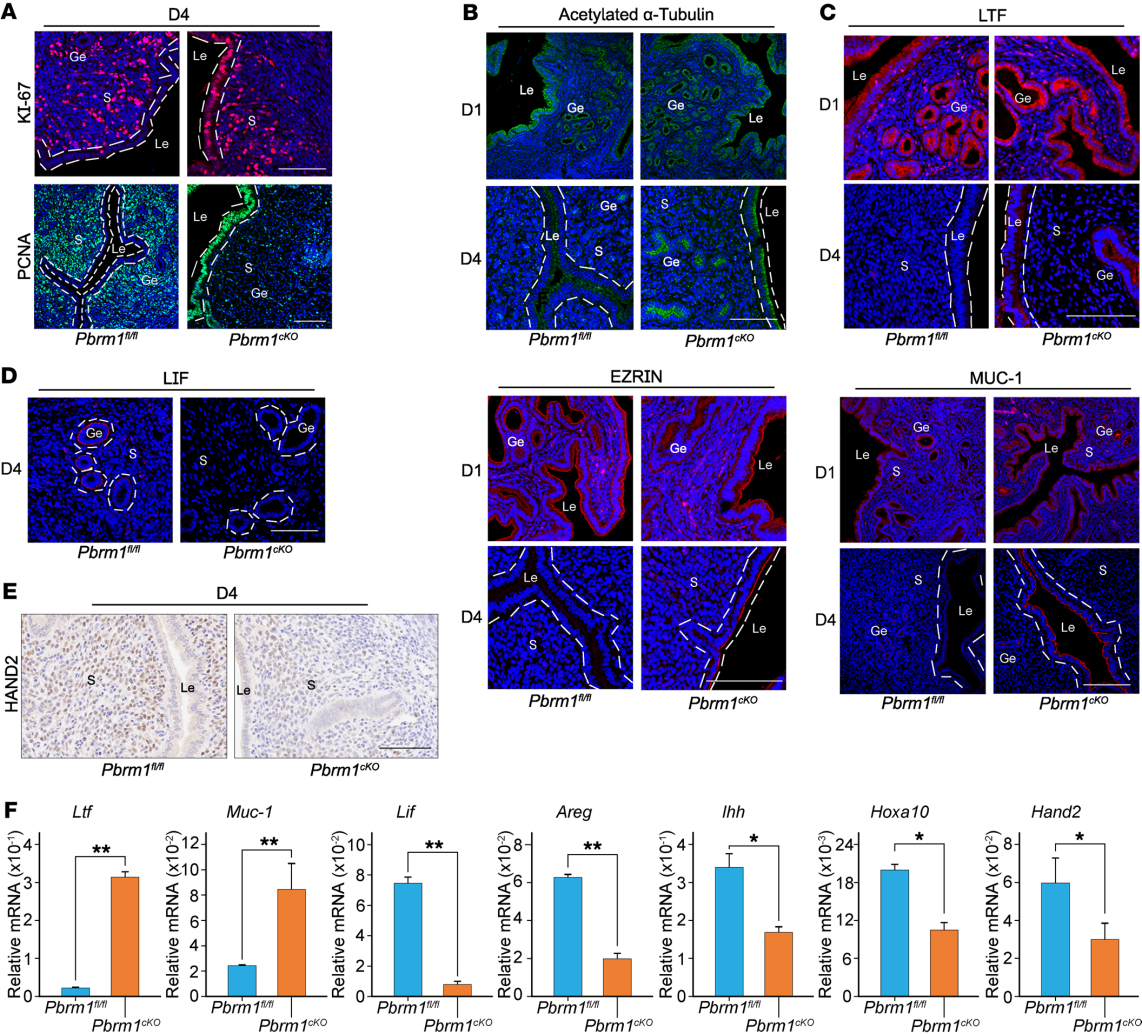
*Pbrm1* deficiency disrupts uterine receptivity and dysregulates E_2_/P_4_ signaling. (**A**) Immunofluorescence staining of KI-67 and PCNA documents an aberrant epithelial proliferation accompanied by a decreased stromal proliferation in *Pbrm1^cKO^* mouse uteri on D4. Scale bars: 100 mm. (**B**) Immunofluorescence staining of cilia marker acetylated α-tubulin (top panels) and microvilli marker EZRIN (bottom panels) in *Pbrm1^fl/fl^* and *Pbrm1^cKO^* mouse uteri. Scale bars: 100 mm. (**C**–**E**) Immunofluorescence (**C** and **D**) and immunohistochemical staining (**E**) of receptivity marker genes document impaired uterine receptivity in *Pbrm1^cKO^* females on D4. Scale bars: 100 mm. (**F**) RT-qPCR analysis of implantation-related marker gene expression in D4 *Pbrm1^fl/fl^* and *Pbrm1^cKO^* uteri. Values are normalized to *Gapdh* expression level and represented as mean ± SEM (*n* = 3). *P < 0.05; **P < 0.01, independent-sample Student’s *t* test.

The uterus is only receptive to blastocyst implantation on D4 (3, 27, 28). Embryo implantation outside of this brief window leads to abnormal pregnancies (3, 24, 28). To understand the underlying pathophysiology of implantation failure in *Pbrm1*-knockout mice, markers of uterine receptivity were analyzed. The failure of the Le to switch from a high to a less apicobasal polar state could cause implantation failure in *Pbrm1^cKO^* mice uterus. We determined cell proliferation and differentiation as defined by KI-67 and proliferating cell nuclear antigen (PCNA) immunostaining, respectively. As illustrated in Figure 2A, the uterine epithelium of *Pbrm1^cKO^* displayed abnormal proliferation accompanied by decreased stromal proliferation on D4. Moreover, the large number of microvilli and cilia in the Le (a signature of polarized cells), as assessed by EZRIN and acetylated α-tubulin, markedly diminishes with implantation (24, 29–31). As shown in Figure 2B, microvilli on the Le surface persisted in D4 mutant uteri, as documented by immunostaining for acetylated α-tubulin and EZRIN. Together these observations suggest impaired uterine epithelial membrane transformation from prereceptive to receptive, independently of the presence of embryos.

Additionally, priming the uterus with P_4_ is obligatory for E_2_ to trigger the uterus to enter a receptive stage in both mice and humans (an evolutionary innovation of eutherian mammals) (1, 14). Attenuation of E_2_-mediated proliferation of uterine epithelia by P_4_ is a prerequisite for successful implantation (13, 32). The expression of uterine receptivity markers responsive to E_2_ include lactotransferrin (*Ltf*), mucin1 (*Muc1*), and leukemia inhibitory factor (*Lif*). Those responsive to P_4_ include amphiregulin (*Areg*), Indian hedgehog (*Ihh*), homeobox A10 (*Hoxa10*), and *Hand2*. Both sets of marker genes were dysregulated in *Pbrm1^cKO^* uteri (Figure 2, C–F). While Lif was normally expressed in the receptive uterine Ge of *Pbrm1^fl/fl^* mice on D4, its expression was completely abolished in the absence of *Pbrm1* (Figure 2, D and F). In contrast, Muc1 and Ltf were abnormally hyperactive in both luminal and glandular epithelial layers of *Pbrm1^cKO^* females on D4 (Figure 2, C and F). Hand2, which is normally expressed in the uterine stroma on D4 and required for stromal-epithelial crosstalk in establishing uterine receptivity, was also abolished in *Pbrm1^cKO^* females (Figure 2, E and F). These abnormal protein expression patterns extended to their cognate mRNAs (Figure 2F). Together, these findings indicate impaired P_4_ gene regulatory activity and loss of the antagonistic influence of P_4_ signaling on E_2_-stimulated epithelial proliferation in *Pbrm1^cKO^* uteri at peri-implantation.

The primary source of serum P_4_ and E_2_ is the ovary. Therefore, we considered the possibility that implantation failure in *Pbrm1^cKO^* females may be due to hormone imbalance, since *PR*^IRES-cre^ expression is present in the corpus luteum of the ovary (Supplemental Figure 1C). However, depletion of luteal *Pbrm1* exerted no apparent influence on expression of P_4_ biosynthesis as reflected by cytochrome P450 cholesterol side-chain cleavage enzyme (P450scc) or 3β-hydroxysteroid dehydrogenase (3β-HSDII) levels (Supplemental Figure 1, C and D). Furthermore, serum levels of E_2_ and P_4_ as well as uterine expression profiles of E_2_ receptor α (*Er*α) and *PR* were comparable in both *Pbrm1^fl/fl^* and *Pbrm1^cKO^* mice (Supplemental Figure 1, E–H). The *PR* promoter–induced cre recombinase is expressed early in the neonate (33). We analyzed the uterine morphology of epithelia, stroma, and myometrium using antibodies to cytokeratin 7 (CK7), FOXA2, and α-SMA as well as apoptosis using CASPASE-3 and γ-H2A. No differences were observed between WT and mutant mice, suggesting that uteri develop normally (Supplemental Figure 1, I and J). Collectively, these observations suggest that impaired P_4_ gene-regulatory defects were not due to shifts in gonadal hormone levels and uterine development.

**Figure 3:**
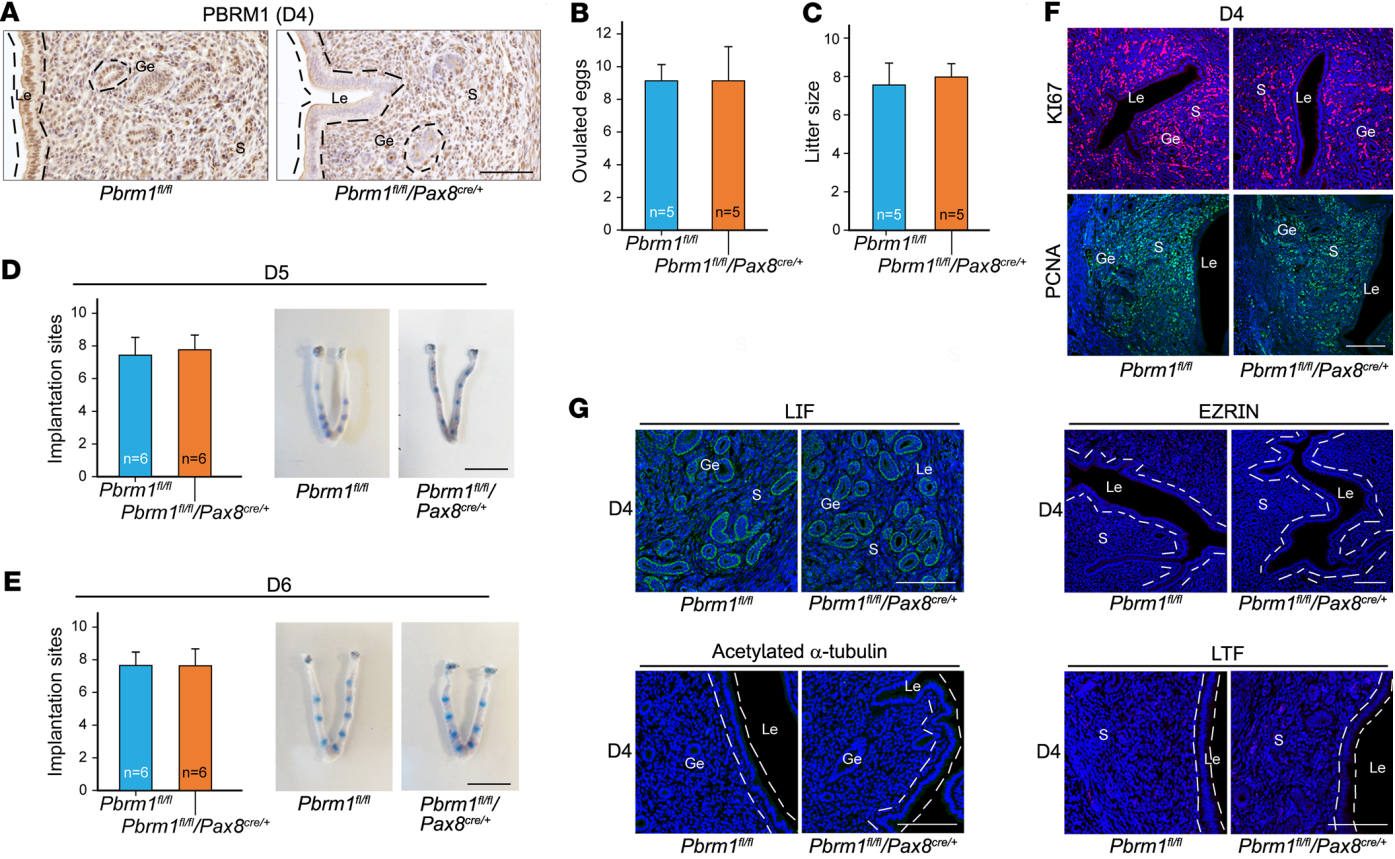
Epithelium-selective deletion of *Pbrm1* leads to normal embryo implantation and female fertility. (**A**) Immunohistochemistry documents specific deficiency of epithelial PBRM1 in *Pbrm1^fl/fl^/Pax8^cre/+^* mouse uteri. Scale bar: 100 mm. (**B** and **C**) Number of ovulated eggs (**B**) and average litter sizes (**C**) of *Pbrm1^fl/fl^* and *Pbrm1^fl/fl^/Pax8^cre/+^* female mice. Numbers within the bars indicate numbers of mice examined. Data are represented as mean ± SEM. (**D** and **E**) Normal implantation in *Pbrm1^fl/fl^/Pax8^cre/+^* mice compared with *Pbrm1^fl/fl^* mice as determined by Chicago Sky Blue dye injection on D5 to D6 of pregnancy. Numbers within the bars indicate numbers of female mice tested. Scale bars: 1 cm. (**F** and **G**) Immunofluorescence images of proliferation status and receptivity marker genes document normal uterine receptivity in *Pbrm1^fl/fl^/Pax8^cre/+^* females on D4. Scale bars: 100 mm.

*Pbrm1* is expressed in both uterine epithelial and stromal cells, and so we sought to ascertain respective contributions to uterine receptivity and implantation. Taking advantage of *Pax8-cre* (*Pax8^cre/+^*) mice, we obtained conditional deletion of *Pbrm1* (*Pbrm1^fl/fl^/Pax8^cre/+^*) specific to uterine epithelium (34). High-knockout efficacy was confirmed by immunohistochemistry (Figure 3A). *Pbrm1^fl/fl^/Pax8^cre/+^* females had normal ovulations and litter sizes as well as typical embryo implantation at D5 and D6 (Figure 3, B–E). Moreover, uterus receptivity, as determined by previously established and related gene markers, did not differ between *Pbrm1^fl/fl^* and *Pbrm1^fl/fl^/Pax8^cre/+^* mice (Figure 3, F and G, and Supplemental Figure 2, A and B). Together, these findings suggest that uterine stromal but not epithelial-expressed *Pbrm1* is essential for normal uterine receptivity.

### PBRM1 activates Hand2 in a SWI/SNF complex–dependent manner.

**Figure 4:**
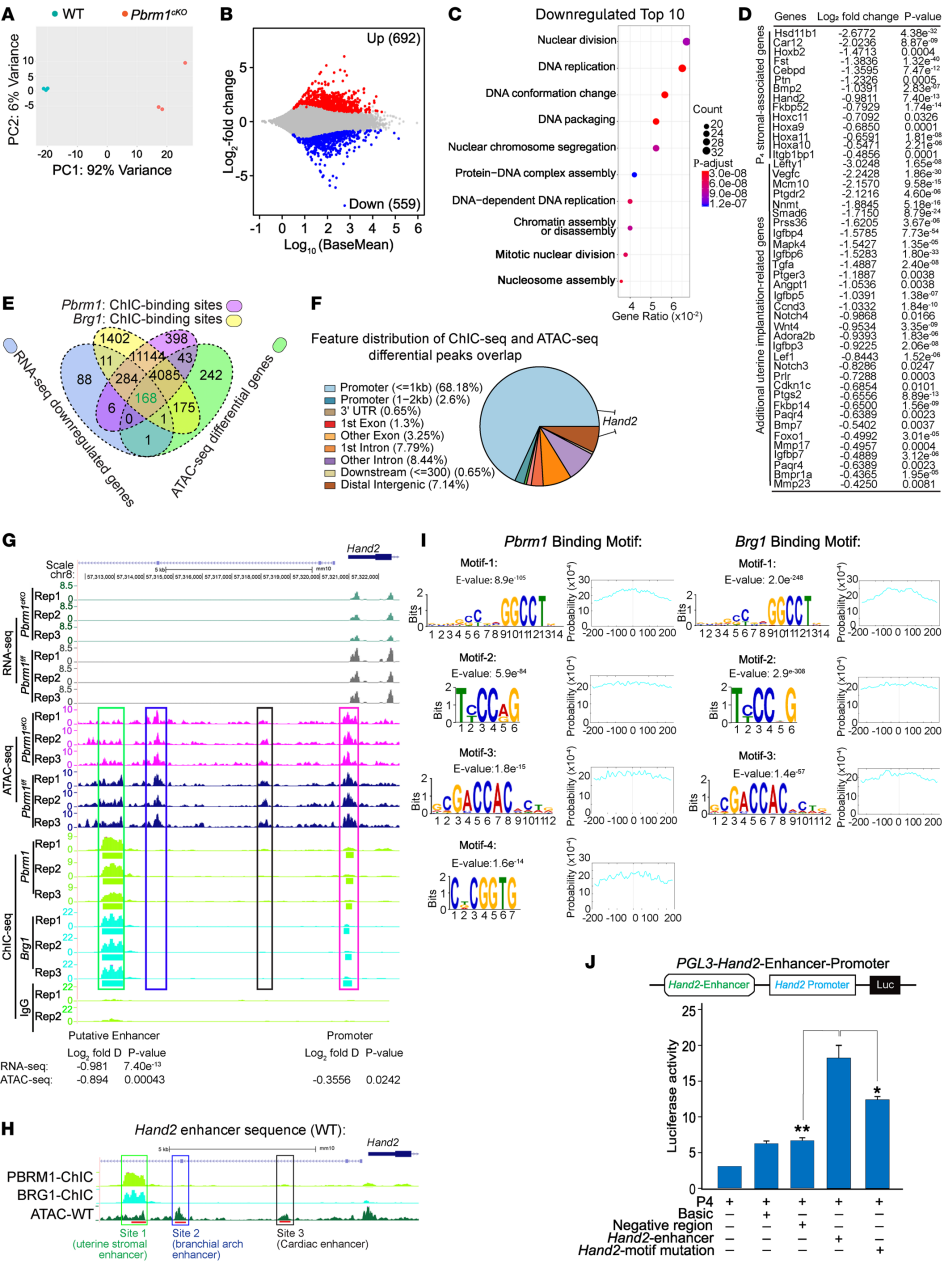
PBRM1 promotion of *Hand2* transcription is dependent on chromatin remodeling. (**A**) PCA plot of RNA-Seq results of uterine stromal cells from *Pbrm1^fl/fl^* and *Pbrm1^cKO^* mice. (**B**) MA plots of differentially expressed RNAs in *Pbrm1^fl/fl^* and *Pbrm1^cKO^* mUSCs. Upregulated and downregulated RNAs are shown as red and blue dots, respectively (>1.5-fold, *P* <0.01, *P*adj < 0.1). (**C**) The top 10 GO terms of downregulated transcripts. (**D**) Genes directly associated with uterine receptivity and implantation that were significantly downregulated (log_2_ fold change and *P* value) from *Pbrm1^fl/fl^* and *Pbrm1^cKO^* uterine stromal cell RNA-Seq. (**E**) Venn diagram showing overlap among genes (*n* = 168) with reduction of RNA expression and differential ATAC accessibility by *Pbrm1* deletion and genes with PBRM1/BRG1 direct binding. (**F**) Genomic distribution of overlapping genes (*n* = 168) that are most likely to be direct targets of the PBRM1/BRG1 complex. (**G**) Genome browser view of normalized RNA-Seq signals, ATAC-Seq, and PBRM1/BRG1 ChIC-Seq tracks for *Hand2* in *Pbrm1^fl/fl^* and *Pbrm1^cKO^* primary mUSCs. Green rectangle, newly identified uterine specific enhancer regulated by SWI/SNF complex; blue rectangle, known branchial arch enhancer; black rectangle, cardiac-specific enhancer; red rectangle, promoter of *Hand2*; Rep 1, 2 and 3, 3 biological replicates. (**H**) Schematic representation of enhancer regions of *Hand2* in different tissues. *Hand2* uterine (site 1, red), branchial arch enhancer (site 2, green), and cardiac enhancer (site 3, blue). (**I**) Prediction of DNA-binding site motifs for PBRM1/BRG1 derived from ChIC-Seq data. Graph to right indicates the probability of the binding motif. (**J**) Renilla-normalized luciferase reporter assay to evaluate *Hand2* promoter activation transfected with *Hand2* WT or motif mutation vectors. Values are represented as mean ± SEM (*n* = 3). *P* values were calculated by post hoc pairwise *t* test after 1-way ANOVA. *P < 0.05; **P < 0.01.

To investigate underlying mechanisms by which *Pbrm1* deficiency decreased uterine receptivity, mouse primary uterine stromal cells (mUSCs) were isolated from *Pbrm1^fl/fl^* and *Pbrm1^cKO^* mice on D4. After differential plating, the purity of stromal cells was verified by vimentin and cytokeratin immunostaining (Supplemental Figure 3, A–C). RNA-Seq analysis identified 692 up- and 559 downregulated genes in *Pbrm1^fl/fl^* compared with *Pbrm1^cKO^* mUSCs (Figure 4, A and B). Downregulated transcripts were enriched (Gene Ontology [GO] analysis) in cell proliferation consistent with aberrant patterns observed in *Pbrm1* mutant uteri. GO analysis also documented decreased chromatin accessibility, consistent with known functions of Pbrm1, a subunit of the SWI/SNF complex (Figure 4C). Some transcripts (e.g., *Ptgs2*, *Bmpr1a*, *Wnt4*) known to be involved in uterine receptivity and embryo implantation, including stromal P_4_-associated genes (e.g., *Hsd11b1*, *Hoxa10*, *Hand2*, *Fkbp52*), were downregulated in *Pbrm1^cKO^* mUSCs (Figure 4D). Together, these data indicated PBRM1 loss has a strong negative impact on normal physiological changes in uterine stromal cells required for embryo implantation.

Assembled SWI/SNF complexes hydrolyze ATP to remodel chromatin. The complex can target distal enhancers to activate proximal promoters of target genes (35, 36). We performed ATAC-Seq to identify potential changes in chromatin accessibility in mUSCs. Although *Pbrm1* deficiency did not disrupt global chromatin accessibility proximal to transcription start sites (TSS) (Supplemental Figure 4, A–D), it did modulate chromatin access for a limited number of cell-specific genes in uterine stromal cells. The predominant genomic distribution of differential ATAC-Seq peaks was positioned at promoter (18.9%) and introns/intergenic (40.24%/31.46%) regions that are the sites of potential enhancers (Supplemental Figure 4, C and D). Pathway analysis (Kyoto Encyclopedia of Genes and Genomes [KEGG]) documented that differential ATAC-Seq downregulated peaks that overlapped with RNA-Seq downregulated genes were enriched with the signaling pathways for MAPK, RAP1, HIPPO, and WNT as well as the cell cycle. These pathways play critical roles in uterine stromal cell proliferation and establishment of embryo receptivity (Supplemental Figure 4E).

We sought to determine whether the presence of PBRM1 in the SWI/SNF complex correlated with specific gene expression in uterine stromal cells and was dependent on BRG1 to remodel chromatin. Therefore, we immunoprecipitated SWI/SNF complexes followed by sequencing (chromatin immunocleavage sequencing [ChIC-Seq]) using antibodies against PBRM1 and BRG1 in WT uterine stromal cells on D4. As indicated in Supplemental Figure 4, F and G, PBRM1 and BRG1 were predominantly positioned at gene promoters and distal intergenic regions (PBRM1 peaks: 38.21%/26.23%, at promoters/intergenic; BRG1 peaks: 30.64%/28.63%, at promoters/intergenic, respectively). To identify potential direct targets responsible for uterine receptivity defects (Figure 4E), we intersected (a) RNA-Seq downregulated genes; (b) ATAC-Seq differential peaks; (c) ChIC-Seq with antibodies to PBRM1; and (d) ChIC-Seq with antibodies to BRG1. More than 90% of PBRM1 peaks overlapped BRG1-binding sites, and we concentrated on the 168 intersecting genes in the 4 data sets. The genomic distribution of PBRM1/BRG1-binding sites and differential ATAC-Seq peaks revealed a high degree of cooccupancy at gene promoter (70.78%) and introns/intergenic regions (24.02%), which are the sites of potential enhancers (Figure 4F).

*Hand2* was identified as a directly regulated gene because of decreased abundance (RNA-Seq) and decreased chromatin accessibility (ATAC-Seq) as well as direct binding by PBRM1/BRG1 (ChIC-Seq) in the promoter region and the adjacent *Hand2os* (opposite strand to *Hand2* locus) site (Figure 4, F and G). Previous studies have demonstrated that *Hand2*, a stromal-specific transcription factor, is an essential regulator of uterine stromal-epithelial crosstalk that establishes uterine receptivity with an identical knockout phenotype to *Pbrm1^cKO^* (13, 14). These observations highlight HAND2 as an appropriate candidate for further investigation in the role of PBRM1 in embryo implantation.

Within 10 kbp upstream of the *Hand2* gene (*Hand2os*) is a region rich in enhancers reported to tightly regulate *Hand2* transcription. Figure 4, G and H, shows the branchial arch–specific enhancer and the cardiac-specific enhancer, both of which are essential for heart development and function (10, 11). ATAC-Seq displayed comparable chromatin accessibility differences in the *Pbrm1* mutation stromal cell at these 2 identified heart *Hand2* enhancer sites. In contrast, chromatin accessibility of this potential enhancer site was reduced in the PBRM1-deficient uterine stromal cells on D4. Interestingly, ChIC-Seq demonstrated substantial binding at what we believe is a newly identified site by PBRM1 and BRG1, but not at the already-reported branchial arch and cardiac-specific enhancer sites (Figure 4, G and H). In addition, the Müllerian duct is the primordial anlage of the female reproductive tract, which differentiates to form the oviduct and uterus (37). Despite the oviduct smooth muscle and uterine stromal cell origin in Müllerian mesenchyme cells, this *Hand2* enhancer region has comparable levels of chromatin accessibility as well as RNA transcriptional activation in conditional null *Pbrm1* and WT primary oviduct smooth muscle cell (Supplemental Figure 5). This suggests that this region is a uterine stromal cell–specific PBRM1/BRG1-regulated enhancer.

To investigate the effect of the SWI/SNF complex on *Hand2* enhancer function, we identified potential binding motifs from PBRM1 and BRG1 ChIC-Seq peaks. We observed that CCAGGGCCT (motif 1), TCCCAG (motif 2), GACCACT (motif 3), and TCGGTG (motif 4) were well conserved and present in the middle of the potential *Hand2* enhancer site (Figure 4I and Supplemental Figure 6, A and B). These 4 motifs were not present in the 2 known heart-specific enhancer regions (Supplemental Figure 6, C and D). To investigate the role of the putative enhancer, we cloned the sequence with either WT or mutated motifs (Supplemental Figure 6, A and B) upstream of a firefly luciferase reporter to assay transcription driven by these constructs or the *Hand2* promoter alone. Our results indicated that the WT enhancer region activates reporter transcription in human uterine stromal cells, but the null mutations of the 4 motifs substantially decreased *Hand2os* enhancer function (Figure 4J).

**Figure 5:**
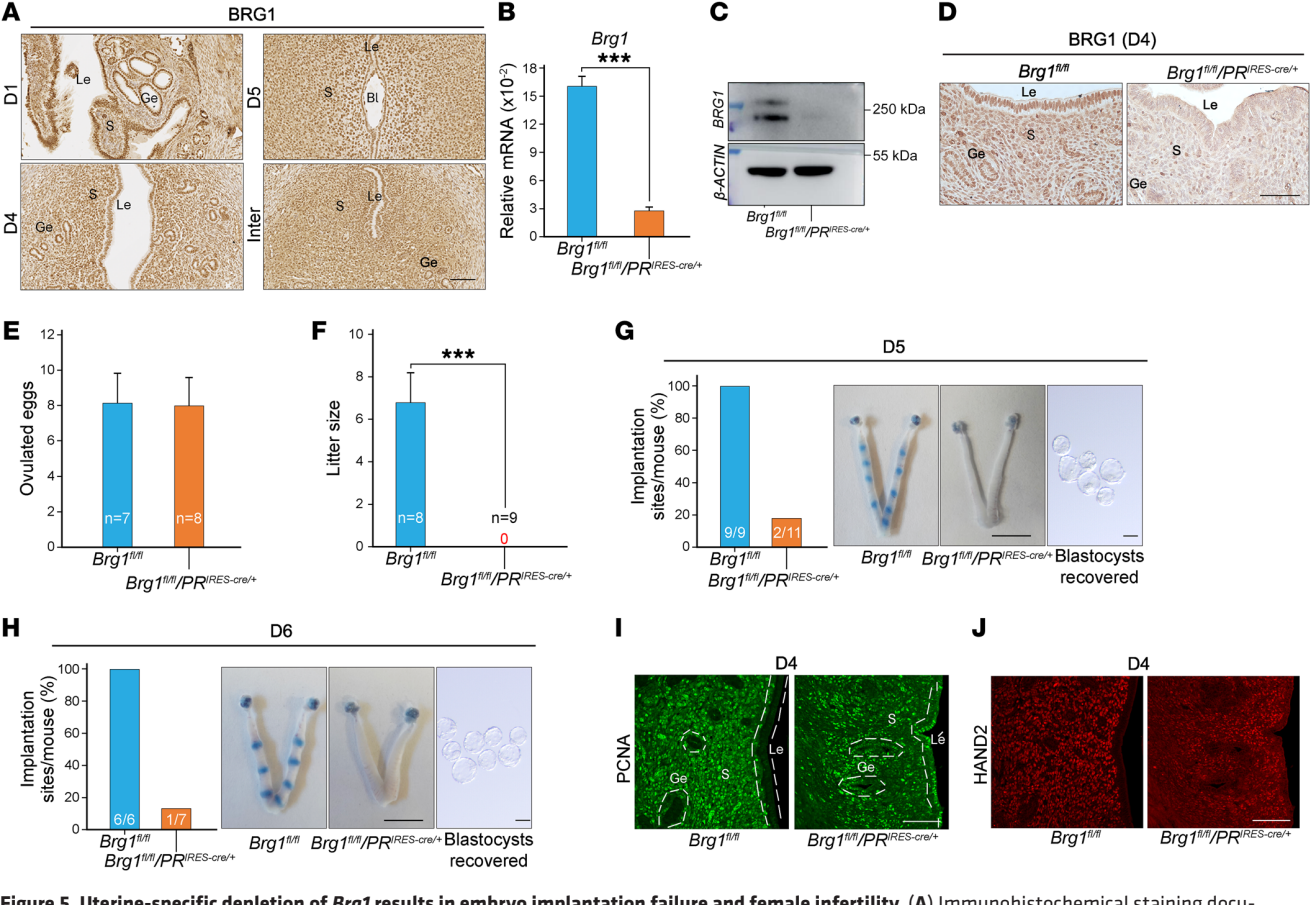
Uterine-specific depletion of *Brg1* results in embryo implantation failure and female infertility. (**A**) Immunohistochemical staining documents a spatiotemporal expression of BRG1 in WT uteri on D1, D4, and D5 of pregnancy. Scale bar: 100 mm. (**B**–**D**) RT-qPCR (**B**), immunoblotting (**C**), and immunohistochemical images (**D**) of *Brg1* mRNA and BRG1 protein levels in D4 uteri from *Brg1^fl/fl^* and *Brg1^fl/fl^/PR^IRES-cre/+^* mice. Values are normalized to *Gapdh* expression and represented as mean ± SEM (*n* = 4). ***P < 0.001, independent-sample Student’s *t* test. β-actin, load control. Scale bar: 100 mm. (**E**) Number of ovulated eggs in *Brg1^fl/fl^* and *Brg1^fl/fl^/PR^IRES-cre/+^* mice. Numbers within the bars indicate numbers of mice tested. (**F**) Average litter sizes of *Brg1^fl/fl^* and *Brg1^fl/fl^/PR^IRES-cre/+^* female mice. Numbers within the bars indicate numbers of mice tested. Data are represented as mean ± SEM. ***P < 0.001, independent-sample Student’s *t* test. (**G** and **H**) Representative images of normal embryo morphology from D5 (**G**) and D6 (**H**) pregnant *Brg1^fl/fl^/PR^IRES-cre/+^* mice, exhibiting embryo implantation failure in the uteri beyond D5. Numbers within the bars indicate numbers of mice with implantation sites per total tested mice. Data are represented as mean ± SEM. **P < 0.01, independent-samples Student’s *t* test. Scale bars, 1 cm (uterus); 100 mm (embryos). (**I** and **J**) Immunofluorescence staining of PCNA and HAND2 document aberrant epithelial proliferation and impaired uterine receptivity in *Brg1^fl/fl^/PR^IRES-cre/+^* females on D4. Scale bars: 100 mm.

*Brg1* encodes a core component of the SWI/SNF complex, which hydrolyzes ATP to remodel chromatin and is expressed in both epithelial and stromal cells at peri-implantation. To ascertain whether PBRM1 modulates the specific enhancer region in a SWI/SNF-dependent manner (Figure 5A), we generated mice with *Brg1* conditionally ablated in PR-positive cells (*Brg1^fl/fl^/PR^IRES-cre/+^*) (Figure 5, B–D). The *Brg1^fl/fl^/PR^IRES-cre/+^* conditional knockout female mice had normal ovulation but were infertile (Figure 5, E and F). No implantation sites were detected in the uterine horns on D5 to D6, and blastocysts were recovered from the conditional null mice because of failure to implant. Embryos in *Brg1^fl/fl^* mice had normal implantation (Figure 5, G and H). As illustrated in Figure 5, I and J, the uterine epithelium of *Brg1^fl/fl^/PR^IRES-cre/+^* mice displayed abnormal proliferation accompanied by substantially decreased expression of *Hand2* in response to P_4_ in stromal cells on D4. The ChIC-binding data indicate multiple cooccupancy of PBRM1 and BRG1 throughout the mouse genome. These observations combined with ChIC-Seq data provide evidence that PBRM1 ensures normal *Hand2* expression by promoter and enhancer chromatin assembly in a SWI/SNF complex–dependent manner.

### PBRM1/BRG1 essential for Hand2 enhancer histone modification/coactivator recruitment.

**Figure 6:**
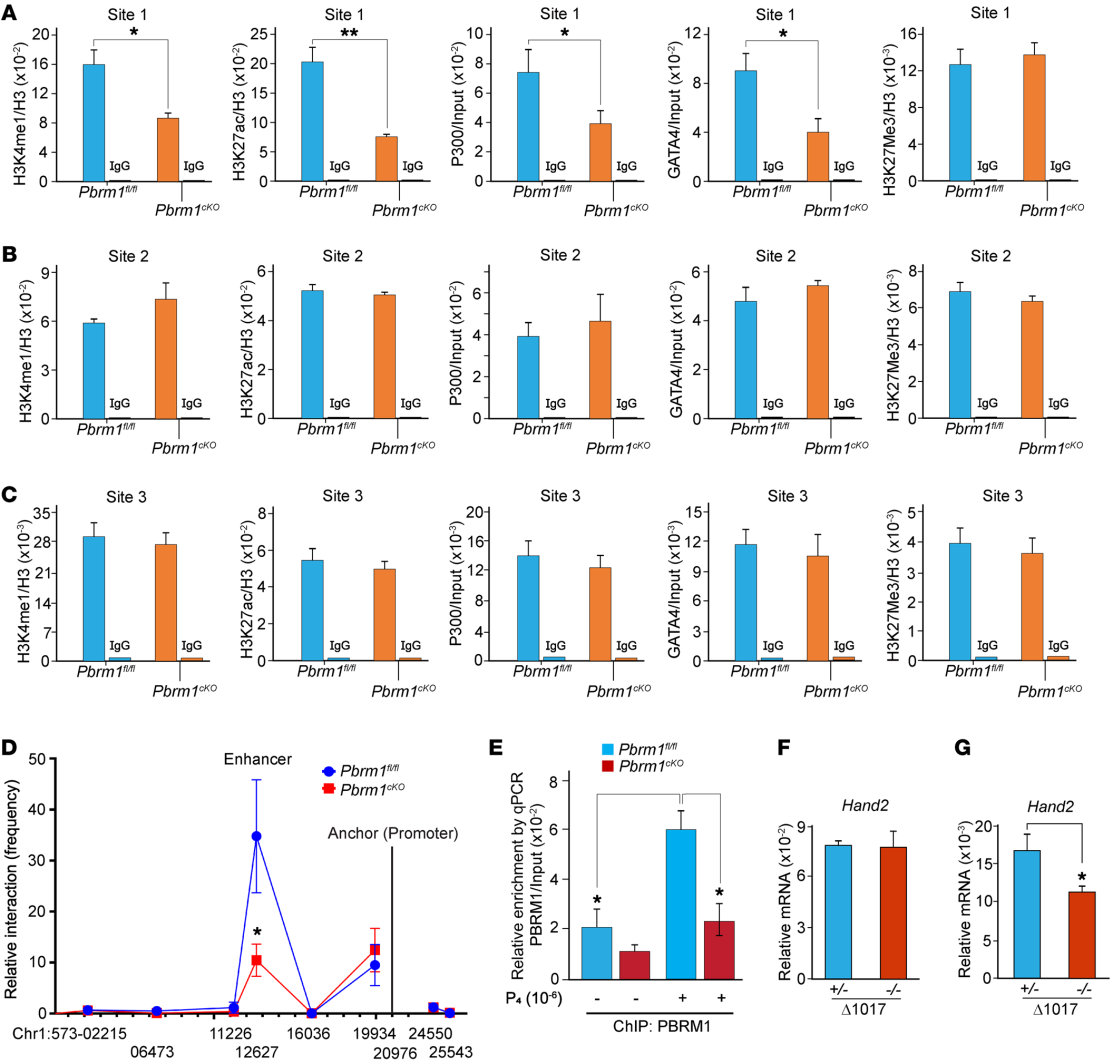
Loss of PBRM1 decreases enhancer histone modifications and recruitment of transcriptional factors and compromises enhancer/promoter interactions. (**A**) ChIP-qPCR for enhancer markers H3K4me1/H3K27ac modifications and recruitment of transcriptional factors *Gata4* and *P300* to the *Hand2* uterine–specific enhancer region (site 1) are reduced in *Pbrm1^cKO^* mUSCs. Values are normalized to input. Data are represented as mean ± SEM. *P < 0.05; **P < 0.01, independent-sample Student’s *t* test. (**B**) The same as **A** of the branchial arch (site 2) and (**C**) cardiac enhancer (site 3) regions showed no differences in the H3K4me1/H3K27ac modifications and transcriptional factor recruitment between *Pbrm1^fl/fl^* and *Pbrm1^cKO^* uterine stromal cells. (**D**) 3C interaction frequency (enhancer-promoter looping) between *Hand2*-specific enhancer and its promoter in *Pbrm1^fl/fl^* and *Pbrm1^cKO^* mUSCs. LCR serves as anchor. Values are represented as mean ± SEM (*n* = 3). *P* values were calculated by Student’s *t* test. *P < 0.05. (**E**) ChIP-qPCR result shows enriched binding of PBRM1 to the putative enhancer site upon P_4_ treatment. *P* value was calculated by post hoc pairwise *t* test after 2-way ANOVA. *P < 0.05. (**F** and **G**) RT-qPCR analysis of oviductal (**F**) and uterine (**G**) *Hand2* transcriptional levels in WT and *Hand2*-specific enhancer knockout mice on D4 of pregnancy. Values are represented as mean ± SEM of 3 biological replicates. *P < 0.05, independent-sample Student’s *t* test.

To further evaluate how compromised enhancer chromatin assembly affects *Hand2* transcription, we used ChIP-qPCR to analyze the newly identified putative site as well as known heart-specific enhancers. Active enhancers are defined by H3K27ac and H3K4Me1 histone marks (22, 23) and are often associated with recruitment of transcription factors GATA4 and P300 (18, 38). In WT uterine stromal cells, we observed both active enhancer marks and recruitment of cofactors, which is consistent with the site being an SWI/SNF-dependent active enhancer. In the absence of PBRM1, there was a substantial reduction of H3K27ac and H3K4me1 and poor recruitment of GATA4 and P300. There were no differences in H3K27me3 repressive marks at this enhancer locus (Figure 6A). We also observed histone modifications (H3K27ac, H3K4Me1) as well as occupancy of transcription factors (GATA4, P300) in the 2 heart-specific enhancers. However, PBRM1/BRG1 did not bind to these regions and there was no comparable reduction of histone marks or transcription factor recruitment in conditional knockout mice (Figure 6, B and C).

ChIC-Seq detected cooccupancy of PBRM1 and BRG1 in the promoter and enhancer regions of *Hand2* (Figure 4, F and G), which suggested that physical interactions (looping) are essential for robust *Hand2* transcription. To investigate this possibility, we used directed chromosome conformation capture (3C) with TaqMan qPCR in WT and *Pbrm1^cKO^* uterine stromal cells. Compared with *Pbrm1^fl/fl^* controls, *Pbrm1* deficiency substantially compromised the enhancer-promoter interactions at the *Hand2* site (Figure 6D). As documented by ChIP-qPCR, P_4_ treatment facilitated PBRM1 binding to this enhancer region and ensured *Hand2* transcription (Figure 6E). In *Pbrm1*-deficient females, exogenous P_4_ supplementation could not restore normal embryo implantation (Supplemental Figure 7 and Supplemental Table 2). To determine whether this uterine SWI/SNF complex functioned as a transcriptional enhancer in vivo, we established *Hand2* enhancer mutant mice with CRISPR/Cas9. After confirmation by DNA sequence, an enhancer deleted mouse line was obtained and designated D1017 (Supplemental Figure 6E) according to the size of the deletion. It is interesting to note the reduced expression levels of *Hand2* in the enhancer mutant mouse uterus, but not oviduct, although both arise from the same embryonic Müllerian duct (Figure 6, F and G).

Taken together, these results substantiate a model that, upon P_4_ priming, the SWI/SNF complex is recruited to an enhancer region of *Hand2* for epigenetic modifications of histones, recruitment of transcription factors, and promotion of enhancer-promoter interactions.

### Increased Fgfs/Fgfr-pErk1/2-pErα signaling in epithelium disrupts embryo implantation.

Stromal, but not epithelial, PBRM1 is critical for transcription of cell-specific *Hand2*. This implies that the uterine stroma must communicate with the Le to affect receptivity for blastocyst implantation. Earlier reports (39–41) suggest that FGFs exert paracrine and juxtacrine responses through cell-surface FGF receptors (FGFRs) and associated docking protein complex. The factors stimulate the receptors to induce phosphorylation of specific tyrosine residues in a critical docking protein, FGFR substrate 2 (FRS2). This induces the coordinated assembly of distinct multiprotein complexes, resulting in activation of extracellular ERK and MAPK signaling cascades (12, 40–42). P_4_-induced expression of *Hand2* in endometrial stroma suppresses multiple FGFs and antagonizes E_2_-induced epithelial gene expression as well as proliferation to promote uterine receptivity. Therefore, in the absence of *Hand2* expression, overactivation of FGFs/FGFRs disrupts stromal-epithelial crosstalk and prevents functional transformation of Le into a receptive state (13).

**Figure 7:**
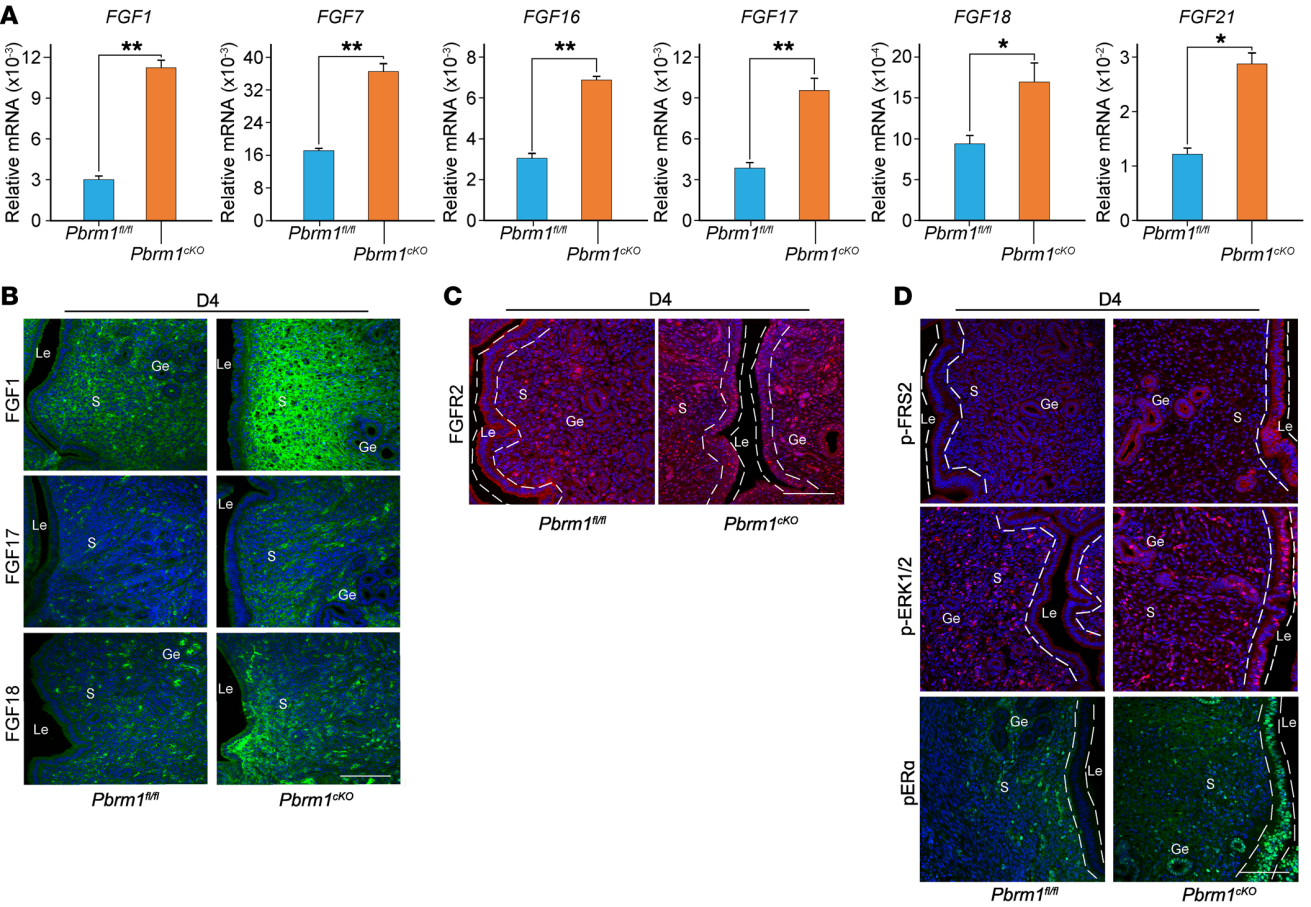
Augmented *Fgfs/Fgfr-pErk1/2-pErα* signaling in *Pbrm1-*deficient uteri disrupts embryo implantation. (**A**) RT-qPCR of FGF family growth factors (*FGF1*, *FGF7*, *FGF16*, *FGF17*, *FGF18*, *FGF21*) in the uterine stroma of *Pbrm1^fl/fl^* and *Pbrm1^cKO^* mice on D4 of pregnancy. Values are normalized to *Gapdh* expression and represented as mean ± SEM (*n* = 3). *P < 0.05; **P < 0.01, independent-sample Student’s *t* test. (**B** and **C**) Immunofluorescence staining of FGFs (FGF1, FGF17, FGF18) and FGFR2 in *Pbrm1^fl/fl^* and *Pbrm1^cKO^* females on D4. (**D**) Immunostaining of p-FRS2, p-ERK1/2, and p-ERα documents augmented *Fgfs/Fgfr-pErk1/2-pEr*α signaling in *Pbrm1^cKO^* Le on D4 of pregnancy. Scale bars: 100 mm.

RNA-Seq differential analysis indicated that mRNA levels of *Fgf1*, *Fgf7*, *Fgf16*, *Fgf17*, *Fgf18*, and *Fgf21* were substantially more abundant in the *Pbrm1*-null uterine stromal cells on D4, which was confirmed by qPCR (Figure 7A). Although limited by the commercial availability of specific antibodies, we observed that FGF1, FGF17, and FGF18 were specifically produced in uterine stromal cells and were markedly upregulated upon loss of *Pbrm1* (Figure 7B). Interestingly, the receptor FGFR2 is also ectopically highly expressed in the epithelial cells of *Pbrm1* conditional null mice (Figure 7C). Activation of the FGF/FGFR signaling pathway in the uterus was further monitored by assessing tyrosine phosphorylation of FRS2 (p-FRS2). Only low expression of p-FRS2 was detected in WT uterine epithelium on D4. In contrast, an increased level was observed in the epithelium of *Pbrm1*-deficient uteri (Figure 7D). We then investigated ERK1/2 signaling downstream of FGFs/FGFRs/p-FRS2 and detected heightened activation of p-ERK1/2 in the epithelium of *Pbrm1* ablated uteri on D4 (Figure 7D). The ERK1/2-dependent phosphorylation of ERα (p-ERα) at position Ser118 in uterine epithelia was present and is required for full activity of the functional E_2_ receptor (ER) (13, 39). Hyperactive p-ERα (Figure 7D), E_2_ regulated gene expression (*Muc1*, *Ltf*), and aberrant cell proliferation in the *Pbrm1*-deficient uterine epithelium (Figure 2, A–D) are consistent with this formulation. Augmented *Fgfs/Fgfr*-*pErk1/2*-*pEr*α signaling in the Le of *Pbrm1*-null uteri facilitates expression of E_2_ response genes. This sustains lengthened microvilli and persistent cell proliferation. The resultant inability of epithelium to transition from high to low apicobasal polarity creates a barrier that further disrupts embryo implantation.

To determine whether the function of PBRM1 is conserved in human uterine stromal cells, we reanalyzed human uterine stromal single-cell RNA-Seq data from women who underwent elective termination of normal pregnancies (age, 25–35 years old; gestational age, 7–9 weeks) without a history of miscarriages (43). As shown in Supplemental Figure 8A, the stromal cells were divided into 2 clusters: one with high and the other with low expression of *HAND2*. Stromal cells with high *HAND2* exhibited high expression of PBRM1 and BRG1, which colocalized with HAND2 in the nuclei of human endometrial stromal cells (HESCs) by immunofluorescence (Supplemental Figure 8B). We constructed knockdown cell lines of *PBRM1* with Tet/on controlled lentivirus-ShRNA which effectively depleted its target in HESC (Supplemental Figure 8, C and D). After P_4_ priming, *PBRM1* deficiency compromised *HAND2* expression compared with controls (Supplemental Figure 8E). This evidence collectively substantiates the potential conservation in HESCs.

## Discussion

Successful implantation depends on a carefully orchestrated dialogue between embryo and hormonally primed maternal endometrium. The process of uterine receptivity is precisely coordinated by ovarian hormones (E_2_, P_4_) that tightly regulate proliferation and differentiation of uterine epithelium and underlying stromal cells. P_4_/PR-regulated genes mediate antiestrogenic signaling and play a critical role in uterine stromal-epithelial communication necessary for successful pregnancy (13). However, the complexity of P_4_/PR-triggered respond genes remain incompletely understood and dynamic changes in transcription factors occupancy, their effect on chromatin structure, and downstream effects on transcription remain to be determined.

Our study includes genetic, biochemical, and bioinformatic evidence of dramatic P_4_ resistance in the absence of uterine PBRM1. This is associated with attenuated stromal growth and heightened epithelial proliferation that we attribute to loss of antagonistic influence of P_4_/PR signaling on E_2_-stimulated epithelium. In turn, this disrupts normal uterine epithelial membrane transformation and creates a barrier so that blastocysts cannot implant. To determine the contribution of epithelial and stromal cell–expressed *Pbrm1* to embryo implantation, we established *Pax8^cre/+^* gene–edited mouse lines. Epithelial deficiency of *Pbrm1* does not compromise uterine P_4_/PR responsiveness, so presumed stromal cell–expressed *Pbrm1* is critical for proper epithelial-stromal crosstalk to establish uterine receptivity and implantation.

**Figure 8:**
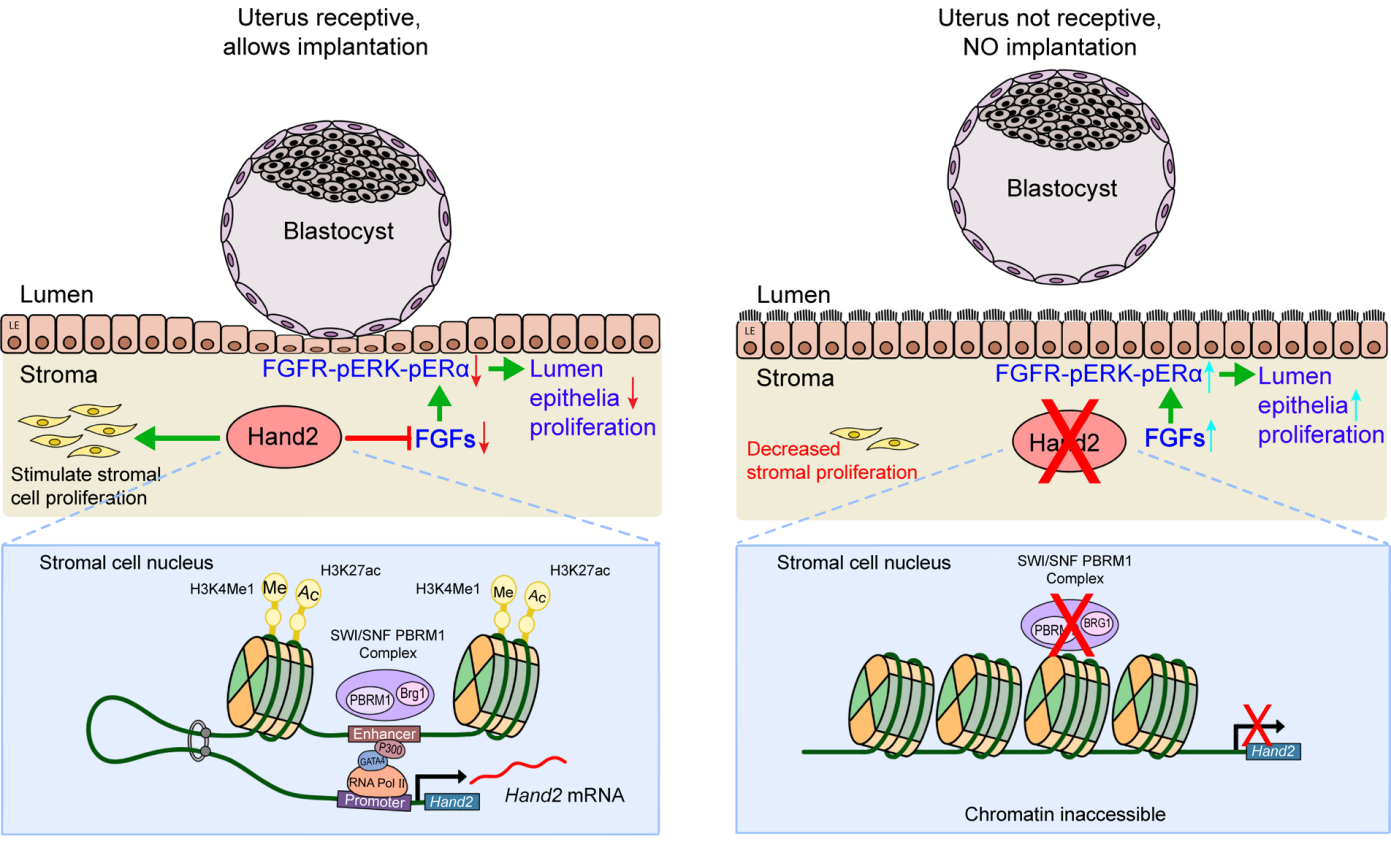
Uterine stromal PBRM1 governs uterine receptivity necessary for embryo implantation. Left: Upon P_4_ priming on D4, PBRM1 is recruited to the *Hand2* enhancer and promoter and facilitates chromatin accessibility and physical interactions (looping) essential for transcription activation in a SWI/SNF complex–dependent manner. This ensures normal stromal-epithelium crosstalk conducive to uterine receptivity and implantation. Right: In the absence of PBRM1 (lower) in the stromal-epithelium, crosstalk does not occur in the absence of *Hand2* expression (upper).

*Hand2*, a bHLH transcription factor involved in cardiac and limb morphogenesis (12, 44), is also expressed in the female reproductive tract exclusively in the uterine stroma (13). In searching for underlying molecular mechanisms, we discovered that uterine stromal cell–expressed PBRM1 binds specifically to the uterine *Hand2* enhancer upon P_4_ stimulation. This is dependent on SWI/SNF chromatin remodeling, which facilitates enhancer accessibility and enables interaction with the promoter. This is essential for optimal stromal cell *Hand2* expression, which instructs P_4_/PR signaling pathways and further antagonizes growth-promoting actions of E_2_. This occurs via stromal cell FGF paracrine signaling acting on the *Fgfs/Fgfr*-*pErk1/2*-*pEr*α pathway in epithelium, ensuring normal stromal-epithelial-embryo dialogue and implantation (Figure 8).

*Hand2* expression is tightly regulated by upstream and downstream enhancers and specific transcription factors in different tissues (10, 11, 45). Located within 10 kbp upstream of the *Hand2* gene is a long noncoding sequence (*Hand2os1*) that is rich in enhancers and tightly associated with *Hand2* transcription. This *Hand2os1* region could contain either negative or positive regulatory elements for expression as well as for subsequent organ development and function (11, 46, 47). Our investigations demonstrate that PBRM1/BRG1 bind selectively to the uterine enhancer/promoters of *Hand2,* but not 2 other known heart enhancers. This binding facilitates chromatin accessibility in a SWI/SNF complex–dependent manner. The putative enhancer was identified based on ATAC-Seq documenting open chromatin accessibility and H3K27ac/H3K4me1 active enhancer histone marks in primary uterine stromal cells. The function of this enhancer was validated by compromised *Hand2* expression after deletion of the genomic sequence–binding site using CRISPR/Cas9. Thus, we provide in vivo genetic evidence that the uterine proximal enhancer is directly bound by the SWI/SNF complex, which loops to the promoter to regulate *Hand2* expression. Motif analysis from PBRM1/BRG1 ChIC-Seq identified 4 potential binding sites near each other and present in the middle of this not-heretofore-described enhancer. In vitro mutations of these binding motifs documented that they are functional for enhancer-promoter activation. Proper chromatin remodeling makes this enhancer more accessible to additional lineage-specific transcription factors (P300, GATA4). Subsequent active histone modification (H3K27ac, H3K4Me1) promotes interaction of enhancer with promoter for optimal *Hand2* transcription (Figure 8).

LncRNA *Hand2os1* transcripts are positioned −123 bp upstream of the TSS of *Hand2* and named upperhand (Uph). They share a promoter and contain 2 conserved *Hand2-*associated heart-specific enhancers within their second intron (9, 11) and 1 uterine specific enhancer within their third intron. Premature transcriptional termination of *Uph* was strongly associated with decreased expression of *Hand2* in heart development, leading to embryonic lethality. This suggests that divergent, noncoding transcription can establish a permissive chromatin environment for specific enhancer activation (11). *Uph* directly interacts with the *Ino80* complex, which is critical for chromatin remodeling. It initiates *Nkx1-2* expression by recruitment of the *Ino80* complex onto its promoter for liver regeneration (48). *Uph* as a P_4_/PR-responsive gene was specifically expressed in stromal cells and strongly associated with decidualization during embryo implantation (49).

*Uph* expression was substantially compromised in *Pbrm1*-deficient uterine stromal cells (our unpublished data). Taken together, these observations could partially explain the increased binding of PBRM1 to the enhancer site in response to P_4_ (Figure 6E). In this scenario, *Uph* expression is elevated after P_4_ stimulation and acts as an adapter to recruit the SWI/SNF complex to enhance *Hand2* transcription. This would lead to epigenetic modification that would facilitate precise temporal and spatial control of *Hand2* expression by P_4_ priming the uterus during embryo implantation. Moreover, after P_4_ priming, posttranscriptional modifications of PBRM1, such as ubiquitination, phosphorylation, and methylation, may be necessary for the recruitment of the SWI/SNF complex to the *Hand2* enhancer and promoter. Genetic ablation of this *Hand2* enhancer deprecates but does not completely inhibit transcription in mUSCs. This is consistent with the observation that *Hand2* expression was not completely abolished in *Pbrm1^cKO^* stromal cells and suggests regulation of *Hand2* expression within the uterus is complicated and nuanced. In addition, since *IHH* signaling also regulates *Hand2* (50), compromised epithelial *Ihh* levels in mutant uteri may also contribute to reduced *Hand2* expression.

Clinically, P_4_ is a key hormone that opposesE_2_-driven growth and differentiation in the endometrium. The suppression of proliferation by P_4_ can disrupt implantation and lead to recurrent miscarriages (24, 32, 51, 52) as well as E_2_-dependent endometrial cancer (53). Insufficient P_4_ resistance is also a hallmark of endometriosis, which is linked to chronic pelvic pain and infertility that affects more than 10% of women of reproductive age. Approximately 1% of cases of endometriosis progress to malignancy (54, 55). Natural and synthetic progestins have been used to treat recurrent idiopathic pregnancy loss, threatened abortion (56), endometriosis (57), and endometrial cancer (58, 59). However, this clinical use is associated with increased resistance to P_4_ and increased predisposition to E_2_-dependent response in endometrium-related diseases. ARID1A can participate as a subunit of the SWI/SNF chromatin-remodeling complex. Its reduction in women with endometriosis is associated with defective implantation and decidualization. These results are attributed to the increased endometrial epithelial proliferation with enhanced E_2_ signaling and attenuation of epithelial PGR (60). Therefore, it is possible that aberrant expression of the SWI/SNF complex could be a potential cause for embryo-implantation failure, unexplained spontaneous miscarriage, and/or endometrial lesions even after P_4_ treatment. Collectively, our investigations of PBRM1 offer a conceptual framework for understanding abnormal endometrial homeostasis and unraveling the nature of these signals, with implications for diagnosis and hormone therapy of nonreceptive endometrium in endometriosis-related infertility.

## Methods

### Mouse strains.

*B6.129P2(Cg)-Pax8^tm1.1(cre)Mbu^/J* (61) and *B6.129S(Cg)-Pgr^tm1. 1(cre)Shah^/J* mice were obtained from the Jackson Laboratory. *Brg1^fl/fl^* mouse lines were provided by Trevor K. Archer (NIH, Durham, North Carolina, USA). A detailed description of these mouse lines was previously published (62, 63). *Pax8^cre/+^* (63) mice were used to establish tissue-specific conditional knockouts of *Pbrm1* in uterine epithelial cell layers. The morning after mating was designated E0.5 or D1 of pregnancy (referring to the embryo or mother, respectively).

### Animal experiments.

*Pbrm1^fl/fl^* or *Pbrm1^cKO^* female mice (≥8 weeks old) were mated with fertile or vasectomized ICR WT male mice to induce pregnancy or pseudopregnancy (vaginal plug, D1 of pregnancy), respectively (24). The next day (D2), oviducts were flushed with KSOM medium (Millipore, catalog MR-101-D; Sigma-Aldrich, catalog MR-101) to recover 2-cell embryos. To visualize embryo implantation at D5 or D6, the number of implantation sites was determined by distinct blue bands after intravenous injection of 0.2 ml 1% Chicago Sky Blue 6B (Alfa Aesar) (28). To examine the effects of ovarian steroids on embryo implantation, mated *Pbrm1^cKO^* female mice with copulation plugs were injected subcutaneously daily (D3–D4) with oil (MilliporeSigma) or P_4_ (2 mg/mouse) (MilliporeSigma). Pregnant mice were sacrificed for analysis on D5, and implantation sites were determined (64). Mice from which no embryos were recovered were excluded from statistical analyses.

### Embryo collection, embryo transfer implantation, and artificial decidualization.

To explore the role of maternal PBRM1 in embryo implantation, we used reciprocal embryo transfer experiments between *Pbrm1^cKO^* and WT mice. After flushing WT ICR female uteri, blastocysts collected in the morning of D4 were transferred into WT or *Pbrm1^cKO^* recipient uteri in the morning of D4 pseudopregnancy. Embryo implantation was assayed on D5 or D6 by intravenous injection of 0.2 ml 1% Chicago Sky Blue 6B dye. Advanced KSOM Medium was used in embryo recovery, transfer, and culture ex vivo. Mice without implantation sites or blastocysts were excluded from statistical analyses (64). All mice were between 2 and 4 months of age. To induce artificial decidualization, 1 uterine horn of D4 pseudopregnant mice was infused with sesame oil (20–25 ml) (MilliporeSigma) and mice were euthanized on D8. The weight of infused and noninfused (control) uterine horns was recorded and served as an index of decidualization (26).

### Generation of Hand2-enhancer knockout mice by CRISPR/Cas9.

The guide RNA sequences (5′-CGCTGAGAGCCCTTTGCACA-3′ and 5′-AATAATTATTGAGCGCAGCT-3′) were designed to target DNA sequences upstream of the *Hand2* start codon. The 2 crRNA solutions were mixed with equal volumes of 200 mM tracrRNA (Integrated DNA Technologies) separately, annealed into crRNA-tracrRNA duplexes (95°C, 5 minutes), and cooled to room temperature; 2 ml of each crRNA-tracrRNA duplex solution was mixed with 1.1 ml *Streptococcus pyrogenes* (S.p.) HiFi Cas9 nuclease (Integrated DNA Technologies) and 48 ml of advanced KSOM Medium to assemble the ribonucleoprotein complex (65). Hormonally stimulated C57LB/6 female mice were mated to C57LB/6 males, and zygotes were collected from oviducts at E0.5 and washed and transferred into advanced KSOM Medium. The ribonucleoprotein complex solutions were mixed and injected into the zygotes in advanced KSOM Medium. Injected zygotes were cultured in KSOM (37°C, 5% CO_2_) to the blastocyst stage. The blastocyst embryos were transferred into the uteri of pseudopregnant ICR female mice on D3.

### Tissue preparation, immunohistochemistry, and immunofluorescence.

Mouse ovary and uteri were fixed in 4% paraformaldehyde (Emsdiasum) overnight at 4°C. Immunohistochemistry and immunofluorescence staining were performed on 5 mm thick, paraffin-embedded sections using antibodies against PBRM1, BRG1, KI-67, PCNA, EZRIN, LTF, MUC1, HAND2, PR, ER, P450scc, hydroxysteroid 11-β dehydrogenase 2 (HSD11b2), CK7, vimentin, and acetylated-tubulin (see Supplemental Table 3). Fluorescent secondary antibodies detected the primary antibody, and DAPI mounting medium identified nuclei. Bright-field images were obtained with an inverted AxioPlan 2 microscope (Carl Zeiss), and fluorescent images were captured with a LSM 780 confocal microscope (Carl Zeiss) (65). Sources of antibodies are listed in Supplemental Table 3.

### Immunoblot.

Protein extraction and immunoblots were performed as described (24). Total protein was extracted in 1× LDS sample buffer with 1× NuPAGE sample reducing agent (Thermo Fisher Scientific). Proteins were separated on 4%–12% Bis-Tris gels and electrophoretically transferred to PVDF membranes (Invitrogen). Signals were detected with PXi Touch (Syngene) or Hyperfilm ECL (GE Healthcare) according to the manufacturer’s instructions. β-Actin served as a loading control. Antibodies are listed in Supplemental Table 3. See unedited blots in Supplemental Figure 9.

### RNA isolation and RT-qPCR.

RT-qPCR was performed as described (24). Total RNA was extracted from uterine tissues or cells using TRIzol reagent (Invitrogen) following the manufacturer’s protocol. SuperScript III First-Strand Synthesis System (Thermo Fisher Scientific) was used for reverse transcription. A total of 3–5 mg RNA was used to synthesize cDNA. RT-qPCR was performed using iTaq Universal SYBR Green Supermix (Bio-Rad) and the QuantStudio 6 Flex Real-Time PCR System (Thermo Fisher Scientific). Expression values were normalized to *Gapdh*, and PCR primers are listed in Supplemental Table 4.

### mUSCs culture.

Uterine stromal cells were isolated and cultured as described (26). Five to ten pseudopregnant D4 *Pbrm1^fl/fl^* and *Pbrm1^cKO^* mouse uterine horns were minced into small pieces (2–3 mm). Tissue pieces were first digested in 5 ml fresh medium (HBSS antibiotic; Gibco) containing 6 mg/ml dispase (Gibco) and 25 mg/ml pancreatin (Sigma-Aldrich) and then incubated in fresh medium (3 ml) containing 0.5 mg/ml collagenase (Sigma-Aldrich) at 37°C for 30 minutes. The digested cells were passed through a 70 mm filter to obtain stromal cells. Cells were plated in 60 mm dishes containing DMEM and Ham F-12 nutrient (F-12) mixture (1:1) (Gibco) with 10% charcoal-stripped FBS (Sigma) and antibiotics (penicillin-streptomycin, Thermo Fisher). After 4 hours, the medium was replaced with fresh medium (DMEM/F-12, 1:1) with 10% FBS. Two hours later, cells were washed twice with PBS for preparation of RNA-Seq, ATAC-Seq, and ChIC-Seq samples. Immunostaining of cytokeratin (1:100; Dako) and vimentin (1:100; Santa Cruz Biotechnology) documented that isolated primary stromal cells were 90%–95% pure.

### RNA-Seq library preparation.

Isolated mUSCs were washed in PBS and kept in RNAlater Stabilization Solution (Thermo Fisher Scientific) at –80°C. Total RNA was isolated using RNeasy Mini or Micro Kit, and mRNA was purified by Dynabeads mRNA Purification Kit (Thermo Fisher Scientific). RNA was quantified with a NanoDrop Spectrophotometer (Thermo Fisher Scientific). First-strand cDNA was synthesized with SuperScript II Reverse Transcriptase (Illumina). For second-strand cDNA synthesis, samples were incubated for 1 hour at 16°C in 55 ml containing 25 ml of the first-strand cDNA synthesis mix, 10 ml resuspension buffer, and 20 ml Second Strand Marking Master Mix (Illumina). The libraries were prepared with a TruSeq RNA Library Preparation Kit (Illumina) per the manufacturer’s protocol in which double-strand cDNA was fragmented, ligated with adapters, and amplified. The final PCR-amplified libraries were pooled and sequenced with single-end 50 bp reads at the NIDDK Genomic Core Facility.

### RNA-Seq data analysis.

Low-quality bases and adaptors were trimmed from sequence reads using cutadapt, version 2.7, with parameters –q 20 -minimum-length 25 -a AGATCGGAAGAGCACACGTCTGAACTCCAGTCA. Resulting reads were mapped to the mouse GRCm38/mm10 genome assembly using HISAT2, version 2.1.0, with default parameters. Aligned reads were counted based on annotation of GENCODE Release m18 using the subread feature Counts, version 1.6.4, with default parameters, except that the “-s” option was used to specify appropriate strands. Differential expression analysis was performed with DESeq2, version 1.22.1, in v3.5.1 R version (66, 67). Functional gene enrichment analysis was performed using clusterProfiler (version 3.10.1) in R (version 3.5.1) on the DE gene list. Genes were tested for enrichment using the GO Biological Process (GO:BP), Cellular Component (GO:CC), and Molecular Function (GO:MF) databases. Dotplot was used to visualized the top 10 significant categories (68).

### ATAC-Seq library preparation.

ATAC-Seq libraries were constructed (69) using 50 to 100 × 10^4^ mUSCs. Cells were centrifuged (500*g*, 5 minutes, 4°C), washed with 200 ml PBS, resuspended in 50 ml cold lysis buffer (10 mM Tris-HCl pH 7.5, 10 mM NaCl, 3 mM MgCl_2_, 0.1% NP-40, 0.1% Tween-20, 0.1% digitonin), and placed on ice. After 3 minutes of incubation, 1 ml of wash buffer (10 mM Tris pH 7.5, 10 mM NaCl, 3 mM MgCl_2_, 0.1% Tween-20) was added. Following centrifugation (500*g*, 10 minutes, 4°C), pelleted nuclei were resuspended in 50 ml of transposition reaction media consisting of 2× tagmentation buffer (25 ml), water (5 ml), PBS (16.5 ml), 10% Tween-20 (0.5 ml), 1% digitonin (0.5 ml),and Tn5 transposase enzyme (2.5 ml, Illumina). After incubation (45 minutes, 37°C) with gentle mixing, DNA was purified using a MinElute PCR Purification Kit (QIAGEN) and eluted into 10 ml of elution buffer. Transposed DNA was amplified using barcoded PCR primers (IDT): 72°C for 5 minutes and 98°C for 30 seconds, followed by 12 cycles of 98°C for 10 seconds, 63°C for 30 seconds, and 72°C for 1 minute. DNA fragments of nucleosome-free regions (between primer dimers and mononucleosome bands) corresponding to sequence inserts of less than 100 bp or more than 1,000 bp were removed from double-sided AMPure XP beads purification and purified using the MinElute Gel Extraction Kit (QIAGEN). Libraries were eluted with 10 ml elution buffer for analysis of size distribution, and 50 bp paired ends were used for sequencing.

### ATAC-Seq data analysis.

Paired-end reads were processed using the ENCODE ATAC-Seq pipeline, version 1.9.0. The genome reference was the mouse GRCm38/mm10 assembly. IDR threshold was 0.05, other parameters were default, and MACS2 was used to call peaks. The IDR conserved peaks were used for further downstream analysis. To identify mutant versus WT differential peaks, DeSeq2 was used to analyze the read counts mapped to the peaks. Peak files for both were merged using BedTools, and resulting bed files defining peaks were assigned to mutant or WT. Reads mapped to these peaks were counted with Feature Counts, version 1.6.4, using default parameters (67). The ATAC-Seq data standards and processing pipeline are at https://www.encodeproject.org/atac-seq).

### Small cell number ChIC-Seq library preparation.

The antibodies to BRG1 and PBRM1 used for ChIC-Seq are listed in Supplemental Table 3. Small cell number ChIC-Seq experiments were performed as previously described with minor modifications (70). Primary mUSCs (1 to 5 × 10^5^) were harvested and crosslinked with 1% formaldehyde (Thermo Fisher) for 5 to 10 minutes at room temperature. Reactions were terminated by adding a 1:10 volume of 1.25 M glycine (MilliporeSigma). For ChIC reaction and library preparation, fixed cells were washed twice with antibody-binding buffer TE (10 mM Tris, 1 mM EDTA, pH 7.5) augmented with 150 mM NaCl and 0.1% Triton X-100 and resuspended (100 ml) in the same. Prebanding 3 ml pAG-MNase and 1 mg antibody were added to samples, mixed, and incubated for 60 minutes at room temperature. After washing 3 times with high-salt buffer (TE, 400 mM NaCl, 1% Triton X-100) and once with rinsing buffer (10 mM Tris-HCl pH 7.5, 10 mM NaCl, 0.1% Triton X-100), 40 ml RSB (20 mM Tris-HCl, pH7.4, 10 mM NaCl, 2 mM CaCl_2_, 0.1% Triton X-100) was added and incubated at 37°C for 3 minutes. The reaction was stopped with 80 ml (20 mM Tris-HCl, pH 8.0, 10 mM EGTA, 20 mM NaCl, 0.2% SDS), and 1 ml Proteinase K (Promega) was added to each sample and incubated at 65°C overnight. DNA was purified by phenol-chloroform extraction. ChIP-enriched DNA was end repaired with the End-It DNA End-Repair Kit (Epicentre), followed by addition of a single A nucleotide and ligation of PE adapters (Illumina). PCR was performed using Phusion High Fidelity PCR Master Mix (New England Biolabs). ChIP libraries were sequenced on Novaseq 6000 (Illumina) SP1 pair-end platforms according to the manufacturer’s protocol.

### ChIC-Seq data analysis.

Paired-end reads were trimmed as previously described (70) and aligned to the mouse GRCm38/mm10 genome assembly using Bowtie2, version 2.3.5. Multimapping reads were removed with samtools, version 1.9, using the “view” subcommand and the additional argument “-q 20” Duplicate reads were removed with the Picard, version 2.21.4, Mark Duplicates tool. BigWig signal tracks were created with deepTools, version 3.3.1, using the bamCoverage tool with the additional arguments “–normalize Using CPM –extend Reads 300”. The mapped, properly paired reads were counted using a script to generate bed files. Peaks were called from these bed files for each replicate individually using MACS, version 2.2.7.1, with the -bedpe option. Pooled peaks were called based on the BAM files of all replicates for a sample (71–73). Motif analysis was performed on 501 bp sequences flanking the peak summits using MEME-ChIP 5.1.0 with standard parameters; JASPAR2018 CORE vertebrates nonredundant and uniprobe mouse were used as known Motif Databases (74). The potential target genes are listed in Supplemental Tables 7 and 8.

### Luciferase assay reporter.

Luciferase assay reporter plasmids of the *Hand2* enhancer-promoter Luc, *Hand2* enhancer (WT and motif mutations), negative control region, and promoter were generated by subcloning genomic DNA PCR products into the pGL-3 promoter vector using the NEB Gibson Assembly System (New England Biolabs). pRL-TK (plasmid-expressing Renilla luciferase, Promega) was cotransfected into the cells to normalize firefly luciferase activity. Commercially available HESCs were derived by introduction of the human telomerase reverse transcriptase (hTERT) into normal HESCs (Kerafast, ENC017). The short tandem repeat (STR) confirmed HESCs were cultured in 24-well plates and subsequently transfected with vectors (total 1.5 mg). The primary cultured cells were maintained in DMEM/F12 without phenol red, 1% penicillin, 1% streptomycin, 1% insulin, transferrin, selenite (ITS), 500 ng/ml puromycin, and 10% charcoal-stripped FBS (CS-FBS) in the presence of P_4_ (10^–7^M). Luciferase activity was measured 48 hours after transfection using a Dual-Luciferase Reporter Assay System (Promega) according to the manufacturer’s instructions. All samples were analyzed in triplicate.

### ChIP-qPCR.

ChIP was performed using the ChIP-IT High Sensitivity Kit (Active Motif) according to the manufacturer’s instructions. Briefly, 0.5 to 1.5 × 10^6^ primary uterine stromal cells were crosslinked with 1% formaldehyde in PBS containing protease inhibitor (Roche) for 15 minutes. After addition of 2.5M glycine (final concentration 0.125M) to stop the reaction, samples were incubated for 5 minutes at room temperature and sonicated to shear DNA into 200 to 800 bp fragments. Crosslinks were enzymatically (Proteinase K, Thermo Fisher) reversed for 3 to 6 hours at 60°C, and DNA was purified by phenol-chloroform extraction. Antibodies used for ChIP are listed in Supplemental Table 3, and the qRT-PCR primers are in Supplemental Table 5. The comparative CT method was applied to calculate the relative enrichment of sequences of interest over H3 and input.

### Lentiviral production and transduction.

Lentivirus was purified as described (25). Using Lipofectamine 2000 (Invitrogen), 293T packaging cells were transfected with 20 mg of lentivirus coexpressing shRNA (shPBRM1#32-Tet-pLKO-puro, shPBRM1#131-Tet-pLKO-puro) or pLKO scramble (75), 15 mg of packaging plasmid psPAX2 (Addgene plasmid 12260), and 5 mg envelope plasmid pMD2.G (Addgene plasmid 12259). Lentiviral supernatants were collected at 40 and 64 hours using a 0.45 mm membrane, pooled, and concentrated using Lenti-X Concentrator (Clontech) following the manufacturer’s instructions. shRNA was introduced into the HESC line, and expression was induced by doxycycline (1 to 2 mg/ml). The knockdown efficiency of hPBRM1 was assessed 5 days after infection, and stable cell lines were validated by immunoblot.

### 3C.

3C assays were performed as previously reported (76) with modifications. From 2 to 8 ×10^6^ mUSCs were crosslinked with 2% formaldehyde in PBS for 15 minutes at room temperature. The reaction was quenched with glycine (final concentration 0.125M). The cells were twice rinsed (10 minutes) with wash buffer (50 mM Tris-HCl, 10 mM EDTA, 0.5 mM EGTA, 0.25% Triton X-100) and stored in buffer (10 mM Tris-HCl, pH 8, 1 mM EDTA, 0.5 mM EGTA) at −80°C. Nuclei were pelleted by centrifugation (6,000*g*, 5 minutes) and washed with the digestion buffer rCutSmart Buffer (New England Biolabs). Nuclei were pelleted again (6,000*g*, 5 minutes) and incubated with digestion buffer containing 0.2% NP-40, 0.1% SDS for 30 minutes at 65°C. Triton X-100 was added to a final concentration of 1%, and samples were further incubated at 37°C for 15 minutes to sequester SDS; 10% of each sample was saved as an undigested control. Next, 2,000 U of *HhaI* (New England Biolabs) was added to a final volume of 100 ml, and the digestion was continued for 1 day at 37°C. The restriction enzyme was inactivated at 65°C for 30 minutes, and another 10% of the sample was saved as a digested control. The digested sample was diluted with 400 ml T4 ligation buffer (New England Biolabs), 1% Triton X-100, and incubated at 37°C for 30 minutes, followed by overnight incubation at 16°C with 4800U T4 DNA ligase (New England Biolabs). DNA was extracted with phenol-chloroform and used as 3C templates for TaqMan-qPCR. The β-actin (Actb) BAC RP23-5J14 (BACPAC) was digested, religated, and used to generate 3C templates to normalize for primer efficiency. Primers targeting the Actb locus were used to equalize loading across samples. A 2-tailed Student’s *t* test was performed with significance defined as *Ρ* < 0.05. Sequences of the 3C primers and loading control primers are included in Supplemental Table 6.

### Statistics.

Unless otherwise noted, statistical analysis was performed with SPSS 11.5. Comparison of means is presented using independent-samples Student’s *t* test. The data are represented as the mean ± SEM. *Ρ* values of less than 0.05 were considered statistically significant.

### Study approval.

All animal studies were performed in accordance with guidelines of the Animal Care and Use Committee of the NIH under a Division of Intramural Research, NIDDK–approved animal study protocol (K018LCDB21, K044LCDB22).

### Data availability.

The next-generation sequencing data in this study have been deposited in the NCBI’s Gene Expression Omnibus database (GEO GSE223711 and GSE229370). All relevant data that support the findings of this study are available upon request. Values for all data points in graphs are reported in the Supporting Data Values file. The Encode pipeline is https://www.encodeproject.org/atac-seq/, https://github.com/lcdb/lcdb-wf

## Author contributions

QX conceived the project. QX and JD designed the experiments. QX and IF performed experiments, and GY provided bioinformatic analyses. QX wrote the manuscript with help from JD. All authors discussed, revised, and approved the manuscript.

## Supplementary Material

Supplemental data

Unedited blot and gel images

Supplemental table 7

Supplemental table 8

Supporting data values

## Version 1 03/01/2024

Electronic publication
