# Supplementary material for: Stromal Pbrm1 mediates chromatin remodeling necessary for embryo implantation in the mouse uterus
Source: J Clin Invest. 2024 Mar 1;134(5):e174194. doi: 10.1172/JCI174194 (PMC10904057; doi:10.1172/JCI174194)
Supplement: Supplemental data [file jci-134-174194-s108.pdf]

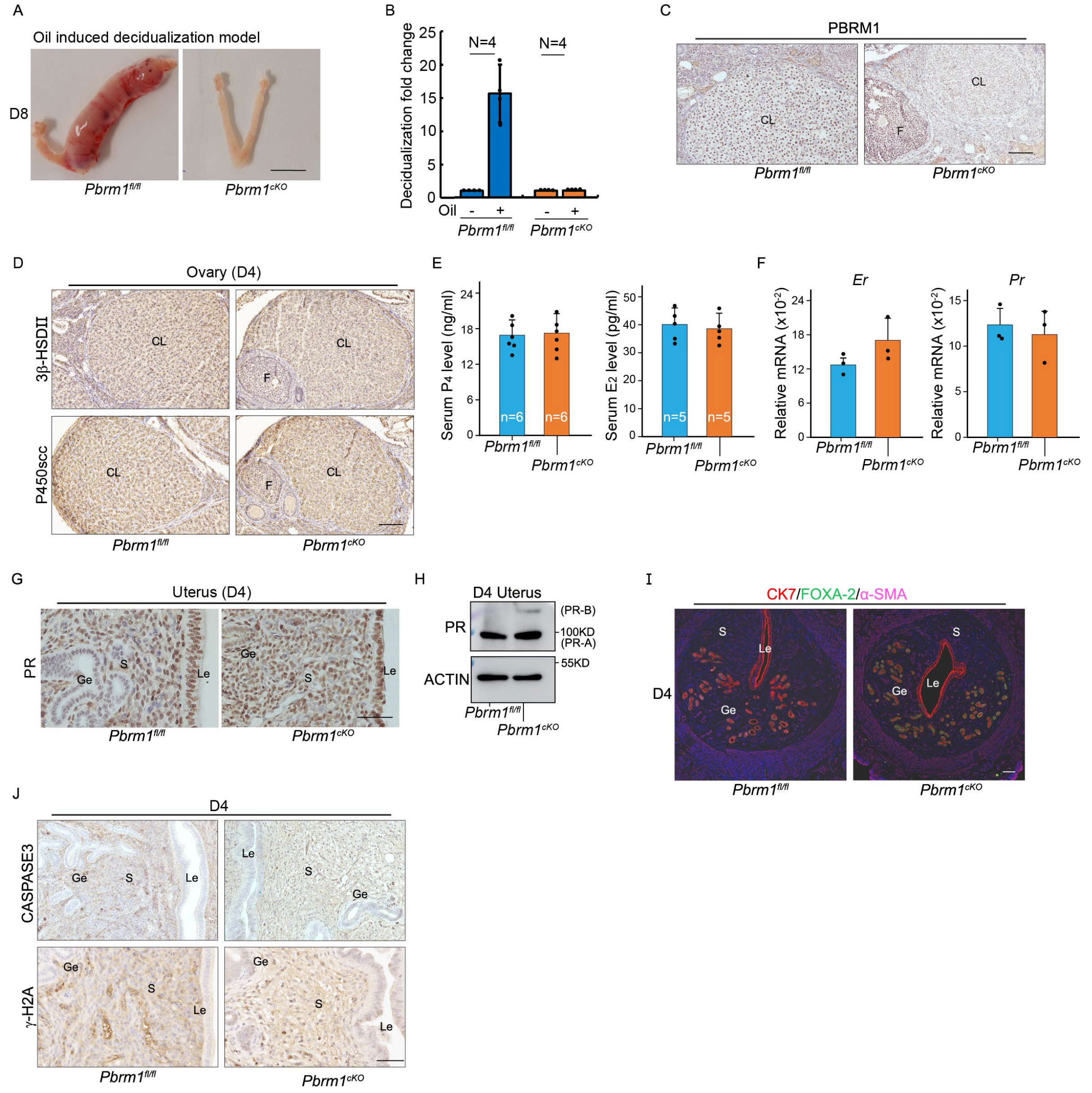

Figure S1. Morphology and ovarian hormones in *Pbrm1$^{fl/fl}$* and *Pbrm1^{cKO}* mice.

(A-B) Artificial decidualization in *Pbrm1$^{fl/fl}$* and *Pbrm1^{cKO}* uteri on pseudopregnant Day 8. *Pbrm1^{cKO}* uterine decidual development in response to oil stimulation is severely impaired. Numbers within the bars indicate the number of mice examined. The *P* value is calculated by post hoc pairwise *t*-test after two-way ANOVA. (C) Immunohistochemistry of PBRM1 in *Pbrm1$^{fl/fl}$* and *Pbrm1^{cKO}* ovaries. CL, corpus luteum; F, follicle. Scale bars, 100 μm. (D) Same as (A) but for 3β-HSDII and P450scc proteins on D4. (E) Comparable serum levels of E_2 and P_4 at D4 in *Pbrm1$^{fl/fl}$* and *Pbrm1^{cKO}* mice. Number within the bar indicates the number of mice tested. Data represent mean ± SEM, independent-samples Student *t*-Test. (F-H) RT-qPCR, immunohistochemistry, and immunoblots of ERα and PR in D4 uteri of *Pbrm1$^{fl/fl}$* and *Pbrm1^{cKO}* mice. Scale bar, 100 μm. Ge, gland epithelium; Le, lumen epithelium; S, stroma. (I) Immunofluorescence staining of CK7, FOXA2 and α-SMA in *Pbrm1$^{fl/fl}$* and *Pbrm1^{cKO}* females on D4. (J) Immunohistochemistry of CASPASE3 and γ-H2A in *Pbrm1$^{fl/fl}$* and *Pbrm1^{cKO}* uterus on D4.

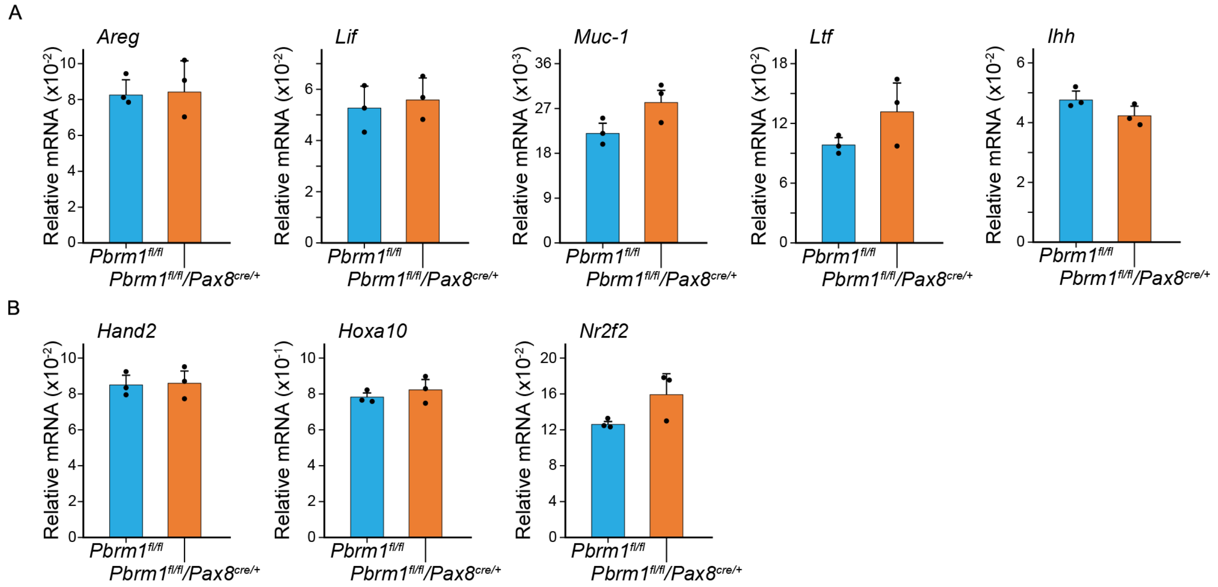

Figure S2. Implantation markers in D4 uteri of *Pbrm1$^{fl/fl}$* and *Pbrm1$^{fl/fl}$/Pax8$^{cre/+}$* mice. (A) RT-qPCR of implantation-related marker genes in epithelium (*Areg*, *Lif*, *Muc1*, *Ltf*, *Ihh*) on D4 in *Pbrm1$^{fl/fl}$* and *Pbrm1$^{fl/fl}$/Pax8$^{cre/+}$* uteri. (B) Same as (A) but for stromal markers (*Hand2*, *Hoxa10*, *Nr2f2*). All values are normalized to *Gapdh* expression and indicated as the mean ± SEM (n=3).

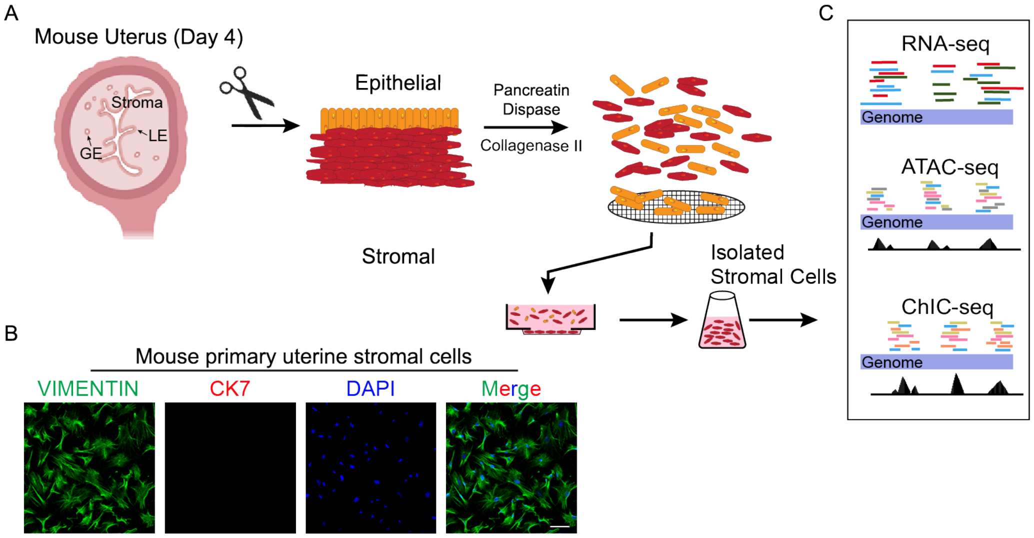

Figure S3. Isolation of primary uterine stromal cells from *Pbrm1*$^{fl/fl}$ and *Pbrm1*^{cKO} female mice.

(A) Primary uterine stromal cells isolated at D4 (10:00 am) from pseudopregnant *Pbrm1*$^{fl/fl}$ and *Pbrm1*^{cKO} mice. (B) Isolated cells are highly enriched for stromal cells as assessed by Vimentin and CK staining. Blue, nuclear staining (DAPI). Scale, 100 µm. (C) Isolated stromal cells were used for RNA-seq, ATAC-seq and ChIC-seq.

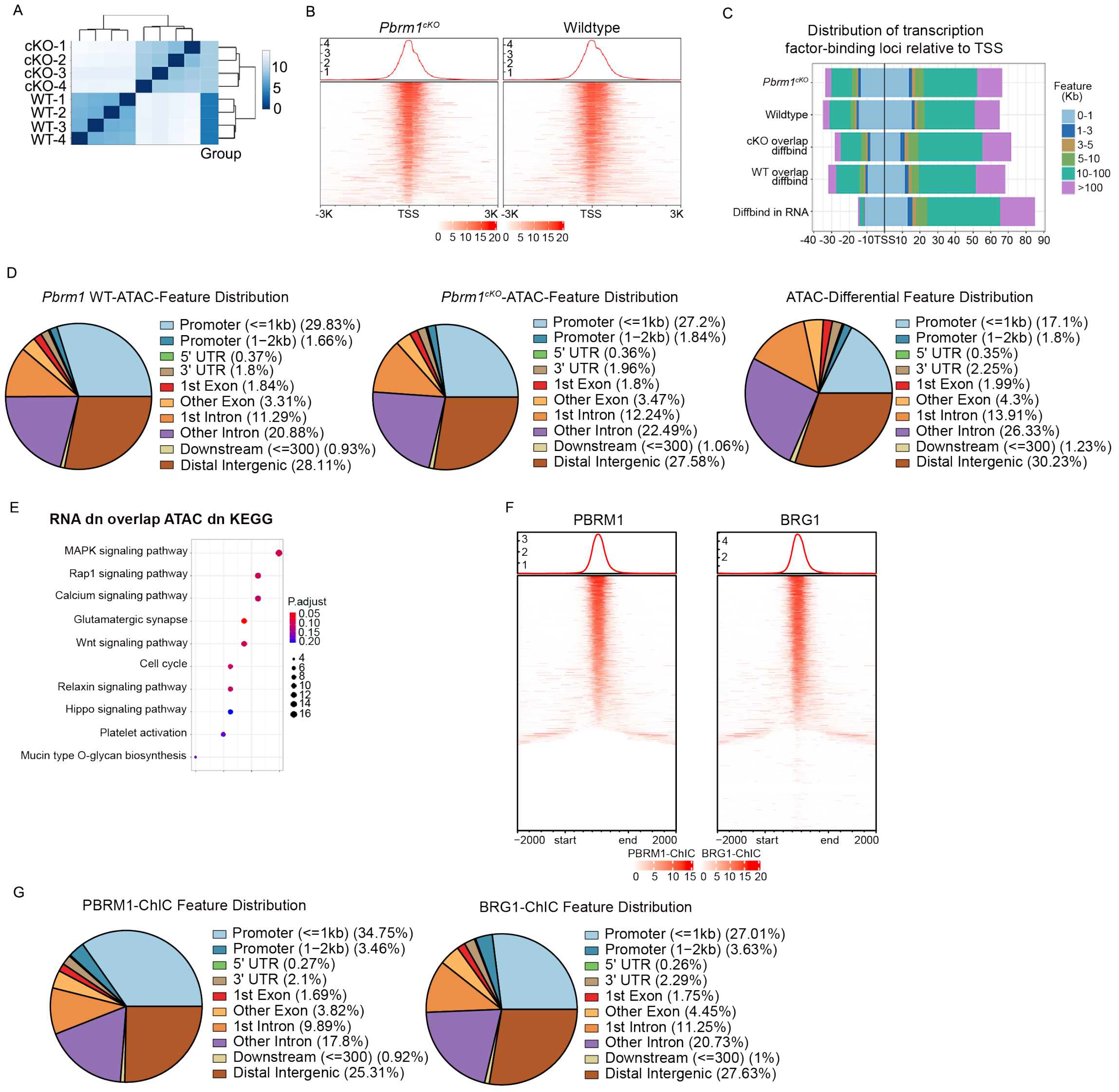

Figure S4. Chromatin accessibility in *Pbrm1*-mutant primary uterine stromal cells. (A) ATAC-seq heatmap of D4 uterine stromal cells from WT and *Pbrm1* deletion female mice. (B) Heatmap showing distribution of WT and *Pbrm1* cKO ATAC-seq peak regions centered at transcriptional start site (TSS). (C-D) Genomic distribution of WT and $Pbrm1^{cKO}$ peaks. (E) KEGG functional analysis for differential peaks in the ATAC-seq down regulated genes (dn) that overlap RNA-seq down regulated genes. (F) Clustered heat maps displaying PBRM1 and BRG1 normalized ChIC-seq peaks centered at the TSS in WT D4 uterine stromal cells. (G) Genomic distribution of PBRM1 and BRG1 ChIC peaks in WT primary uterine stromal cell on D4.

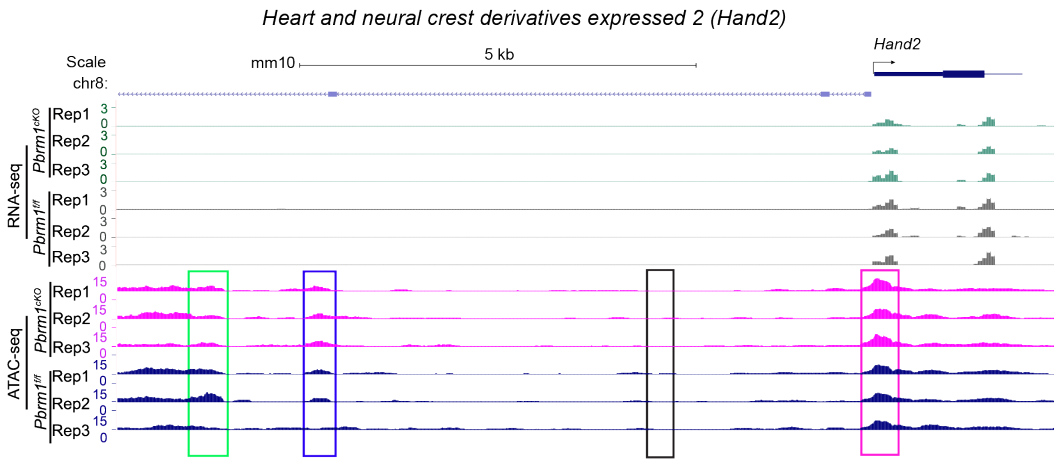

Figure S5. Comparable *Hand2* transcription level and chromatin accessibility in *Pbrm1$^{fl/fl}$* and *Pbrm1^{cKO}* primary mice oviductal smooth muscle cells.
Genome browser view of normalized RNA-seq signals and ATAC-seq tracks for *Hand2* in *Pbrm1$^{fl/fl}$* and *Pbrm1^{cKO}* primary mice oviductal smooth muscle cell. Green rectangle, a newly identified uterine specific enhancer regulated by SWI/SNF complex. Blue rectangle, known branchial arch enhancer. Black rectangle, cardiac-specific enhancer. Red rectangle, promoter of *Hand2* directly bound by the SWI/SNF complex. Rep 1, 2 and 3, three biological replicates.

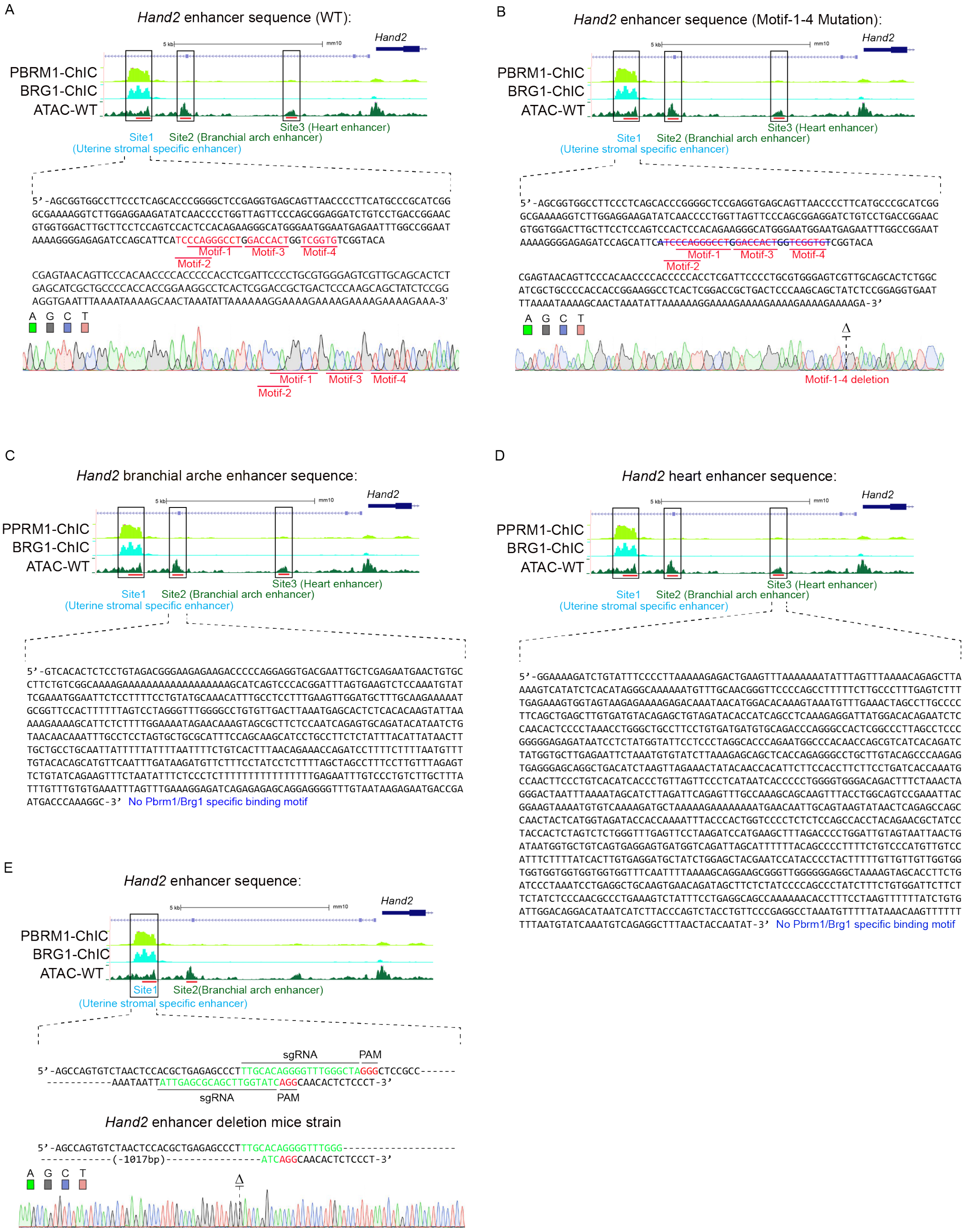

Figure S6. Predicted binding motifs in the *Hand2* gene locus and uterine specific enhancer knockout mice generated with CRISPR/Cas9.
(A-B) Predicted binding motifs in the middle of putative uterine specific *Hand2* enhancer region. Luciferase constructs with the putative *Hand2* WT (A) and mutated motifs (B) enhancer region. The red highlights motifs 1-4. (C-D) The *Hand2* heart and branchial arch specific enhancers did not have the PBRM1/BRG1 specific binding motifs. (E) Design of the putative uterine specific *Hand2* enhancer region and DNA sequence at the enhancer locus of 2 knockout mouse lines.

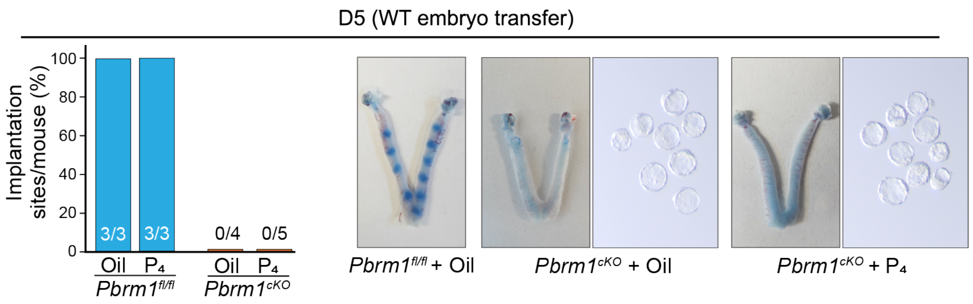

Figure S7. Exogenous progesterone supplementation cannot improve embryo implantation in *Pbrm1* mutant females.

Implantation rate and representative morphology of uteri from *Pbrm1*$^{fl/fl}$ and *Pbrm1*^{cKO} female mice treated with oil or P_4. Number within the bar indicates the number of mice with implantation sites per total tested mice.

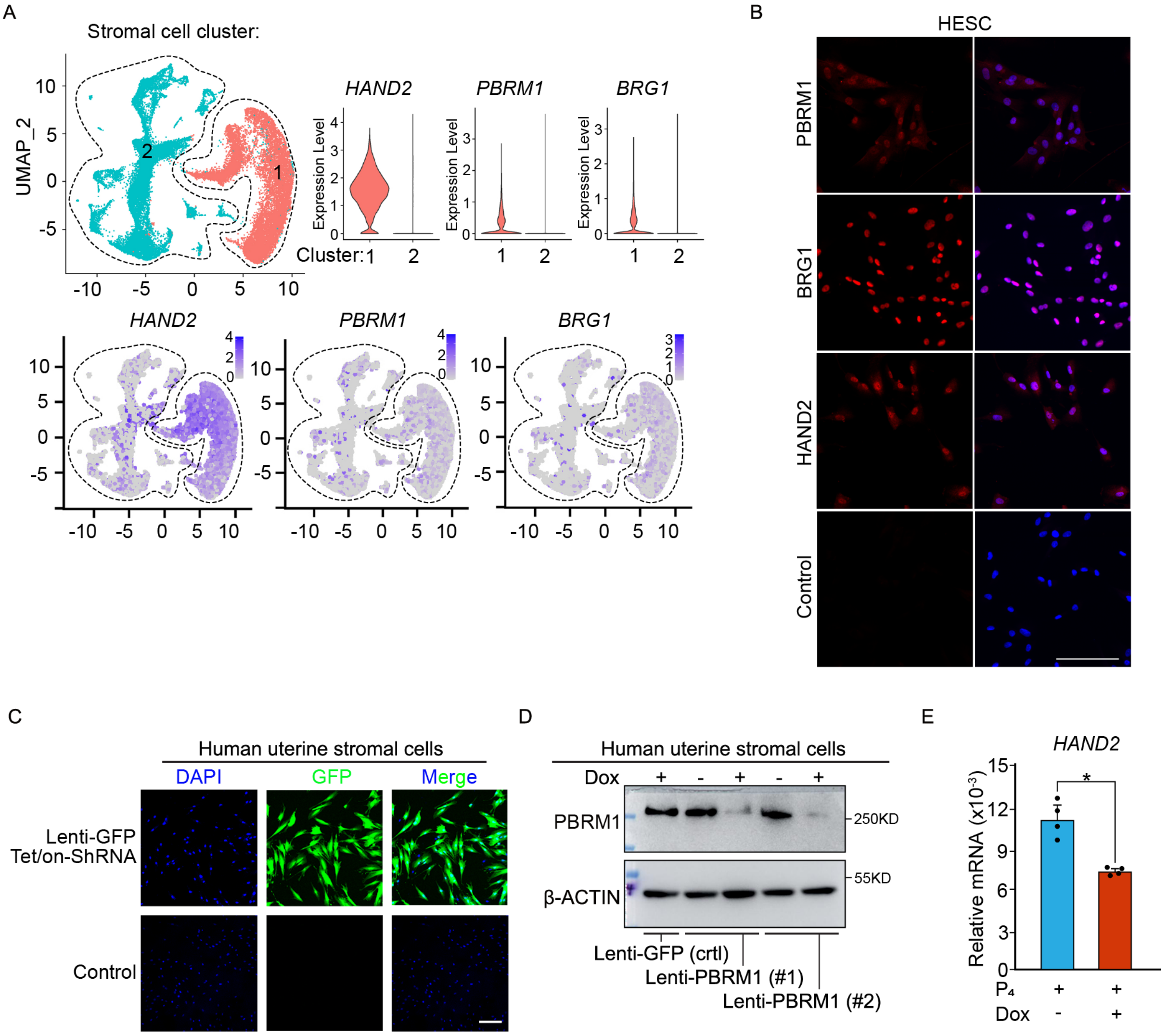

Figure S8. *PBRM1*, *BRG1* and *HAND2* expression in human uterine stromal cells. (A) Single cell RNA-seq documents co-expression of *PBRM1*, *BRG1* and *HAND2* in normal pregnant women uterine stromal cells (data from GSE194219). (B) Immunostaining of PBRM1, BRG1 and HAND2 demonstrates co-localization in the nuclei of human uterine stromal cells. (C) Confocal images of GFP documents transduction efficiency after lentivirus infection of human uterine stromal cells. Scale bar: 100 µm. (D) Immunoblot analysis of PBRM1 protein expression in human uterine stromal cells. β-actin, loading control. (E) Quantitative real-time PCR of *HAND2* in *PBRM1* knockdown of human uterine stromal cells. Data shown represent the mean ± SEM. *P<0.05, independent-sample Student's t test.

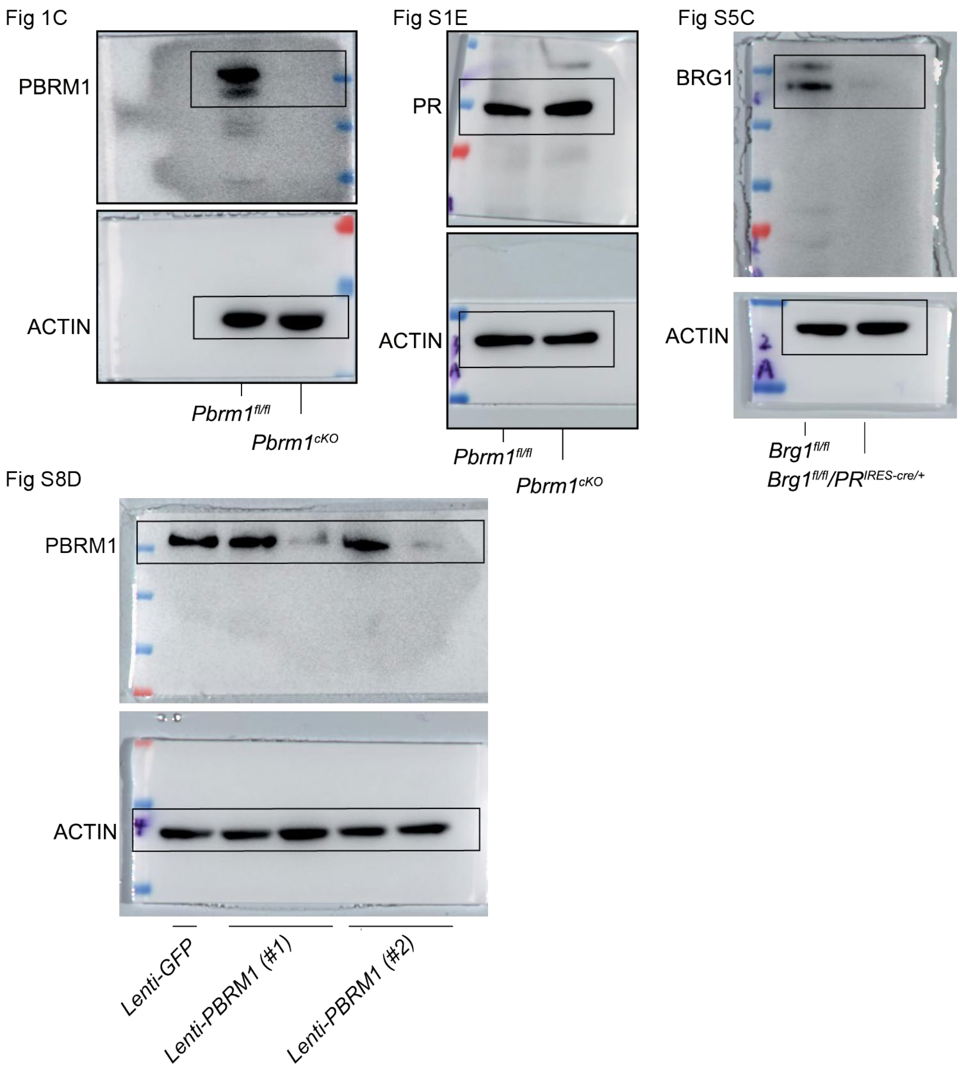

Figure S9: Images of uncropped immunoblots.

Supplemental Table 1: Reciprocal embryo transfers between *Pbrm1*$^{fl/fl}$ and *Pbrm1*^{cKO} mice

Genotype		Recipients	Embryos transferred	Mice with IS (%)	IS (%)	No Sites (%)	Embryos recovered
Embryo	Recipient						
WT	*Pbrm1*$^{fl/fl}$	3	45	3 (100%)	32 (71%)	0	0
WT	*Pbrm1*^{cKO}	8	128	1 (12.5%)	2 (1.5%)	7 (87.5%)	51 (40%)

Blastocysts were collected by flushing uterus on D4 of pregnancy and transferred into recipient uterus on D4 (9:00) of pseudopregnancy. Implantation sites were visualized by the blue dye in the midmorning (10:00 h) of D5 of pregnancy. IS, implantation sites; WT, wildtype.

Supplemental Table 2: Exogenous P_4 supplementation cannot improve normal embryo implantation in *Pbrm1* mutant females

Genotype		Treatment	Recipients	Embryos transferred	Mice with IS (%)	IS (%)	No IS (%)	Embryos recovered
Embryo	Recipient							
WT	*Pbrm1*$^{fl/fl}$	Oil	3	42	3 (100%)	31 (74%)	0	0
		P_4	3	42	3 (100%)	29 (69%)	0	0
WT	*Pbrm1*^{cKO}	Oil	4	60	0	0	4 (100%)	23 (38%)
		P_4	5	77	0	0	5 (100%)	32 (41%)

Pregnant *Pbrm1*$^{fl/fl}$ and *Pbrm1*^{cKO} mice were injected subcutaneously with oil or progesterone (P_4, 2 mg/mouse) on D3. Blastocysts were collected by flushing uterus on D4 WT of pregnancy and transferred into recipient uterus on D4 (9:00) of pseudopregnancy. Implantation sites were visualized by the blue dye in the midmorning (10:00 h) of D5 of pregnancy. IS, implantation sites; WT, wildtype.

Supplemental Table 3: Antibodies

Antibodies	Company	Catalog	Dilution	Application
BRG1	Abcam	Ab11064	1:300	Immunohistochemistry
PPRM1	Abcam	Ab196022	1:500	Immunohistochemistry
P450scc	American Research	35-152123	1:100	Immunohistochemistry
HAND-2	R&D	AF3876	1:100	Immunohistochemistry
HAND-2	Abcam	Ab200040	1:800	Immunohistochemistry
PBRM1	Bethyl	591A-T	1:200	Immunohistochemistry
ERα	AB Clonal	A12976	1:300	Immunohistochemistry
PR	Cell Signaling	#8757	1:300	Immunohistochemistry
11β-HSD2	Santa Cruz	Sc-365529	1:50	Immunohistochemistry
FGFR1	Cell Signaling	9740T	1:300	Immunohistochemistry
BRG1	Cell Signaling	3508T	1:200	Immunohistochemistry
BRG1	Thermo Fisher	720129	1:200	Immunohistochemistry
FGFR2	Thermo Fisher	PA5-14651	1:200	Immunofluorescence
α-SMA	R&D	MAB1420	1:200	Immunofluorescence
LTF	Bioss	51024M	1:200	Immunofluorescence
CK	Dako	M351529-2	1:200	Immunofluorescence
LIF	Novusbio	39N7D10	1:200	Immunofluorescence
Mucin-1	Abcam	Ab15481	1:200	Immunofluorescence
Acetylated tubulin	Sigma-Aldrich	T7451	1:500	Immunofluorescence
Ezrin	Cell Signaling	#3145	1:200	Immunofluorescence
Vimentin	R&D	MAB2105	1:100	Immunofluorescence
KI-67	Abcam	15580	1:500	Immunofluorescence
PCNA	Santa Cruz	Sc-56	1:300	Immunofluorescence
p-ERa(Ser-118)	Santa Cruz	SC-12915	1:100	Immunofluorescence
p-FRS2(Y436)	R&D	AF5126-SP	1:200	Immunofluorescence
p-ERK(E-4)	Santa Cruz	7383	1:200	Immunofluorescence
p-ERK1/2 (Thr177/Thr160)	Covalab	00120243	1:200	Immunofluorescence
FGFR2	Thermo Fisher	PA5-14651	1:60	Immunofluorescence
HAND-2	Abcam	Ab200040	1:800	Immunofluorescence
FGF17	Sigma-Aldrich	HPA052600	1:300	Immunofluorescence
FGF1	Novus	2E12	1:200	Immunofluorescence
PBRM1	Bethyl	591A-T	1:1000	Immunoblot
BRG1	Abcam	Ab11064	1:1000	Immunoblot
PBRM1	Bethyl	590A-T	1:1000	Immunoblot

Supplemental Table 4: Primers for real-time PCR

Pbrm1 Mus RT-F	5'-GGTGAAGAAGGAATGATGGAAGACATG-3'
Pbrm1 Mus RT-R	5'-GGGAGAAGCCATGTCATCATCATCA-3'
Lif Mus RT-F	5'-TCTATGGTTCCAGGCCTTTCC-3'
Lif Mus RT-R	5'-CTATGGTTCCAGGCCTTTCCTAA-3'
Hand2 Mus RT-F	5'-TCGGTTATCTAGTGCTGTC-3'
Hand2 Mus RT-R	5'-ATACTTACAATGTTTACACCTTCA-3'
Muc1 Mus RT-F	5'-AGCCCCTATGAGGAGGTTTCG-3'
Muc1 Mus RT-R	5'-AAGTGGTCACCACAGCTGGG-3'
Ltf Mus RT-F	5'-GGGCAAGTGCGGTTTAGTT-3'
Ltf Mus RT-R	5'-CCATTGCTTTGGAGGATTT-3'
Areg Mus RT-F	5'-GACAAGAAAATGGGACTGTGC-3'
Areg Mus RT-R	5'-GGCTTGGCAATGATTCAACT-3'
Hoxa10 Mus RT-F	5'-GGCAGTTCCAAAGGCGAAAA-3'
Hoxa10 Mus RT-R	5'-CAAAAAAAGCCAGAACAAAC-3'
Brg1 Mus RT-F	5'-AGTACATGATTGTGGATGAAGGCCAC-3'
Brg1 Mus RT-R	5'-GGTGCATTGAACCACTGTTCGAAG-3'
Ihh Mus RT-F	5'-CATCTTCAAGGACGAGGAGAACA-3'
Ihh Mus RT-R	5'-CATGACAGAGATGGCCAGTGA-3'
Gapdh Mus RT-F	5'-TGGCAAAGTGGAGATTGTTGCC-3'
Gapdh Mus RT-R	5'-AAGATGGTGATGGGCTTCCCG-3'
ER Mus RT-F	5'-AATGATGGGCTTATTGACCAACCTA-3'
ER Mus RT-R	5'-CCTTCCACACATTTACCTTGATTCC-3'
PR Mus RT-F	5'-ACCTGATCTAATCCTAAATGA-3'
PR Mus RT-R	5'-ATTGTGTTAAGAAGTAGTAAGAC-3'
Fgf1 Mus RT-F	5'-ATCACAACCTTCGCAGCCCTG-3'
Fgf1 Mus RT-R	5'-CTGAGCTGCAGCTGAATGTGCT-3'
Fgf7 Mus RT-F	5'-ACCTCGTCTGTCTAGTGGGCACT-3'
Fgf7 Mus RT-R	5'-TACCACTGGGTGCGACAGAACA-3'
Fgf16 Mus RT-F	5'-GTTTCCTGAACGAGCGCCTGG-3'
Fgf16 Mus RT-R	5'-GTGCCGTTGGGGAAGATCTCAA-3'
Fgf17 Mus RT-F	5'-AGCAGGCGGCAAATCCGTGAATA-3'
Fgf17 Mus RT-R	5'-ACAGATGTACTTCTCGCTCTCTGCC-3'
Fgf18 Mus RT-F	5'-AAGCAGCTGCGCTTGTACCAG-3'
Fgf18 Mus RT-R	5'-TGATCCGGACTTGACTCCCGAAG-3'
Fgf21 Mus RT-F	5'-ATCTAGAGTTGGGACCCTGGGACT-3'
Fgf21 Mus RT-R	5'-ATCTCCAGGTGGGCTTCAGTGTC-3'

*F, forward primer; R, reverse primer

Supplemental Table 5: Primers for ChIP-qPCR

Hand2 ChIP Site1-F	5'-AGAGATCCAGCATTCATCCCAGGG-3'
Hand2 ChIP Site1-R	5'-AGTTGCTTTTATTTTAAATTCACCTCCGGAG-3'
Hand2 ChIP Site2-F	5'-GGCAGGTTGATCTAGTCTTGAGCC-3'
Hand2 ChIP Site2-R	5'-CAGATTAGACCCCAGGGAAAAGAGC-3'
Hand2 ChIP Site3-F	5'-GAAACTAGCCTTGCCCCTTC-3'
Hand2 ChIP Site3-R	5'-GGGTGCCTAGGGAGGAATAC-3'

*F, forward primer; R, reverse primer

Supplemental Table 6: Primers for chromosome conformation capture (3C)

Hand2-neg 3C-57302215	5'-CCTTTGAGCTCGTCTTGACTTTGA-3'
Hand2-neg 3C-57306473	5'-GATCACTGTGAGTTCCAGGCCA-3'
Hand2-neg 3C-57311226	5'-ACATAAGAAGCGTGCGTGGGTAT-3'
Hand2-neg 3C-57312627	5'-TCGCTTCCACCAATGGCTCTG-3'
Hand2-neg 3C-57316036	5'-TGCTTAAATCCACTGCTAGCTAGTTCC-3'
Hand2-neg 3C-57319934	5'-GTGGAGATCCATTTTCCGTTCTAATGC-3'
Hand2-neg 3C-57324550	5'-CCTAATCTTTACATGGGTGATACCTCAAA-3'
Hand2-promoter 3C-57320976	5'-TGTACAGCTACATCTTTAGGGCCG-3'
Hand2-enhancer 3C- 57312627	5'-CAGAGCCATTGGTGGAAGCGA-3'
Actb 3C site-1	5'-GCAAGTGCTTCTAGGCGGACTGTTA-3'
Actb 3C site-2	5'-CCTGTGGTTGTCAGAGCAACCTTCTA-3'
Actb 3C site-3	5'-CCCCGAGGTGACTATAGCCTTCTTTT-3'
Actb 3C site 4	5'-CCATCTTGAGAGTACACAGTATTGGGAACC-3'
