# Supplementary figures and images for: Stromal Pbrm1 mediates chromatin remodeling necessary for embryo implantation in the mouse uterus
Source: J Clin Invest. 2024 Mar 1;134(5):e174194. doi: 10.1172/JCI174194 (PMC10904057; doi:10.1172/JCI174194)

Fig 1C

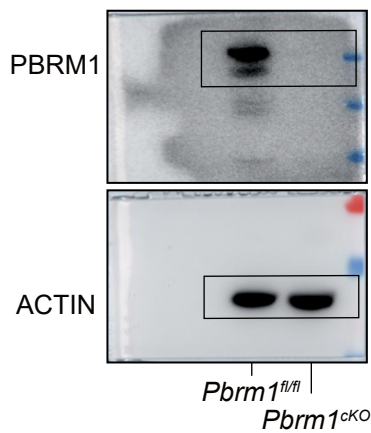

Fig S1E

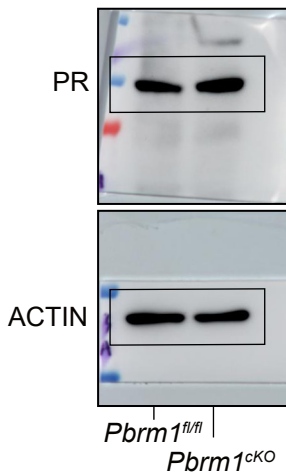

Fig S5C

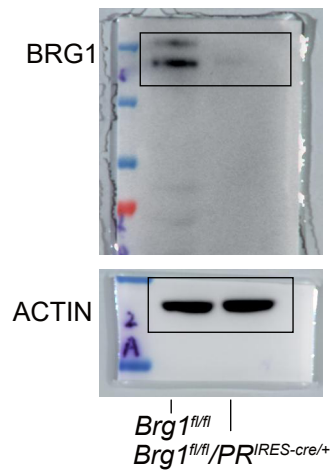

Fig S8D

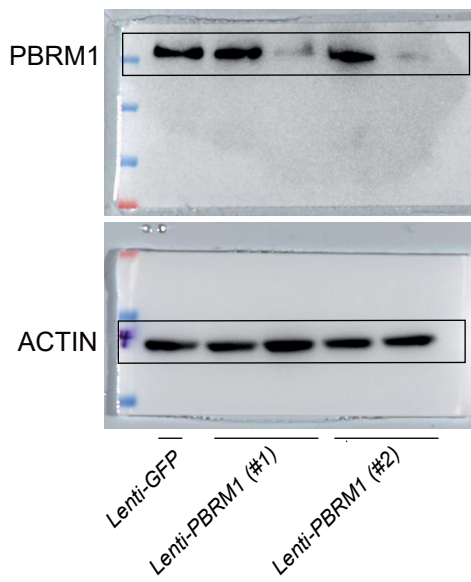

Supplement: Unedited blot and gel images [file jci-134-174194-s109.pdf]
